# Clinico-genomic nomograms for estimation of survival risk in metastatic castrate-resistant prostate cancer

**DOI:** 10.1093/jncics/pkag042

**Published:** 2026-06-03

**Authors:** Manish Kohli, Anushka Shankar, Jennifer Lloyd, Liang Wang, Xingyue Huo, Muhammad Zaki Fadlullah, Aik Choon Tan, Joseph Finkelstein

**Affiliations:** Division of Oncology, Department of Medicine, Huntsman Cancer Institute, University of Utah, Salt Lake City, UT, United States; Division of Oncology, Department of Medicine, Huntsman Cancer Institute, University of Utah, Salt Lake City, UT, United States; College of Nursing, University of Utah, Salt Lake City, UT, United States; Department of Tumor Biology, H. Lee Moffitt Cancer Center, Tampa, FL, United States; Arizona Center for Telemedicine and Digital Health, Department of Medicine, Division of General Internal Medicine, Geriatrics and Palliative Medicine, College of Medicine - Tucson, University of Arizona, Tucson, AZ, United States; Department of Oncological Sciences, Huntsman Cancer Institute, University of Utah, Salt Lake City, UT, United States; Department of Oncological Sciences, Huntsman Cancer Institute, University of Utah, Salt Lake City, UT, United States; Arizona Center for Telemedicine and Digital Health, Department of Medicine, Division of General Internal Medicine, Geriatrics and Palliative Medicine, College of Medicine - Tucson, University of Arizona, Tucson, AZ, United States

## Abstract

**Background:**

To enhance risk stratification in metastatic castrate-resistant prostate cancer (mCRPC), we developed clinical and integrated clinico-genomic prognostic nomograms combining clinical prognostic factors with copy number alteration-based risk scores (RSs) in circulating tumor DNA (ctDNA) and metastatic tissue to predict 1-, 2-, and 3-year overall survival (OS) probabilities.

**Methods:**

A clinical prognostic nomogram was developed from publicly accessed Yale University Open Data Access (YODA) data-sciences project (*N* = 1088 patients). A second publicly available independent dataset (*N* = 82) with an 11-gene prognostic RS developed concurrently obtained ctDNA and matched metastatic tissue specimens (gains: AR, MYC, COL22A1, PIK3CA, PIK3CB, NOTCH1; losses: TMPRSS2, NCOR1, ZBTB18, TP53, NKX3-1) was used to develop an integrated clinico-genomic survival nomogram. Independent predictors for survival were identified in each dataset using univariate and multivariate Cox proportional hazard. Multivariate regression coefficients with statistical significance (*P* < .05) were used to develop prognostic nomograms to estimate 1-, 2- and 3-year OS probabilities. Nomogram performances were assessed using time-dependent area under curves (t-AUCs). All analyses were conducted in RStudio (v4.1.2).

**Results:**

The YODA dataset t-AUCs for the prognostic nomogram for estimating OS at 1, 2, and 3 years were 0.74, 0.70, and 0.74. In the independent second dataset, the integrated clinico-genomic prognostic nomogram was observed to have higher t-AUCs for predicting 3-year mCRPC survival at 0.807 compared with 0.714 using clinical factors alone.

**Conclusions:**

An integrated clinico-genomic nomogram with ctDNA RSs achieved the highest t-AUC for 3-year survival in mCRPC and may enable precision in identification of poor-prognosis subgroups based on somatic alterations.

## Introduction

Prostate cancer is projected to cause more than 35 770 deaths among US males in 2025^1^ and more than 325 000 deaths globally,^2^ with most cancer-related mortality occurring in the metastatic castrate-resistant prostate cancer (mCRPC) state. Intensification of androgen deprivation therapy (ADT)–based regimens in managing metastatic hormone-sensitive prostate cancer (mHSPC) has slowed disease progression to mCRPC state^3^ but there is inevitable progression. In mCRPC, the overall survival (OS) is heterogenous with a median of 34 months,^4^ and prognostic survival models for mCRPC are based on protein levels of serum alkaline phosphatase (ALP), serum lactate dehydrogenase (LDH), serum albumin, and hemoglobin^5-7^ along with clinical factors of performance status and opioid use.^8^^,^^9^ The clinical prognostic models were validated in 8083 chemotherapy-naive mCRPC patients enrolled in 7 randomized controlled phase 3 trials^8^ and a 2-risk (low and high) and a 3-risk prognostic classifier (low, intermediate, and high) was developed. Importantly, the clinical risk classifiers may facilitate more balanced patient allocation to trial arms helping mitigate survival bias associated with unequally distribution of baseline prognostic risk across treatment arms. Clinical factor–based risk groups, however, fail to estimate individual mCRPC patient survival probabilities in clinical practice in a patient-centric approach. Incorporating clinical, laboratory risk factors into prognostic survival nomograms can overcome this limitation. Table S1 lists 20 mCRPC studies with patient-centric nomograms or risk-group-based mCRPC risk stratification models using clinical factors, in relatively small patient cohorts that include a mixed population of mCRPC patients before and after initiating mCRPC-specific treatments.

We accessed a large, publicly available treatment-naïve mCRPC dataset that had previously been used to define mCRPC prognostic risk groups,^8^^,^^10^ and we applied it to develop patient-centric nomograms estimating 1-, 2-, and 3-year survival probabilities based on clinical variables. While clinical, nontumor biology–based nomograms (Table S1) can assist with individualized survival estimation, they limit the advancement of precision medicine approaches for patients whose genomic alterations are associated with poor survival and who could benefit from genotype-directed therapies if molecular prognostic factors are included in survival estimations. Molecular prognostic factors in mCRPC include plasma cell-free DNA alterations such as circulating tumor DNA (ctDNA),^11^  *TP53* mutations, *AR* amplifications, *RB1* loss,^11^^,^^12^ and other genomic tissue–based prognostic classifiers.^11-16^ Additionally, plasma cell-free microRNA (miR) profiling (elevated miR-375; miR-1290) has prognostic relevance in mCRPC.^14^^,^^17^^,^^18^ Unlike in other tumor types,^19^ clinico-genomic prognostication has not been well elucidated in mCRPC. In this study, in addition to developing clinical factor–based survival nomograms in mCRPC, we integrated mCRPC genomic and clinical prognostic factors for estimating individual patient-centric survival using a publicly available matched concurrently obtained metastatic tissue-liquid biopsy biospecimen mCRPC treatment-naïve dataset.

## Methods

Prognostic nomograms for mCRPC survival were developed using 2 independent treatment-naïve mCRPC datasets to estimate probabilities for 1-, 2-, and 3-year OS. A clinical factor–based nomogram was developed from publicly accessed Yale University Open Data Access (YODA) data-sciences project, which includes patient variables and survival outcomes of 1088 patients enrolled in the “COU-AA-302” study.^4^^,^^9^^,^^10^ University of Utah institutional review board (IRB) exemption for research was obtained prior to research analysis (IRB-00186890) and no patient contact was made on the deidentified data. For developing an integrated clinico-genomic prognostic nomogram, we accessed a second publicly available independent dataset of a prospective cohort study in treatment-naïve mCRPC patients called “PROMOTE,” which has previously reported an mCRPC prognostic risk score (RS) from copy number alterations (CNAs) in 11 genes.^12^ In this dataset, we also compared the performance of the integrated clinico-genomic prognostic nomogram with clinical factor–based nomogram alone. A detailed description of the 2 treatment-naïve mCRPC datasets is provided in Supplementary Methods.

Briefly, the “COU-AA-302” study randomized patients to receive abiraterone acetate/prednisone (“AA/P”) (study arm) vs prednisone (“P”) alone (control arm). A total of 846 patients were included in the analysis, with 462 in the study arm and 384 in the control arm and the YODA dataset was accessed for clinical factors and survival outcomes. Details for mCRPC patient eligibility and recruitment for the single-arm prospective cohort, “PROMOTE” (*Pro*state Cancer *M*edically-*O*ptimized Genome-Enhanced *T*h*e*rapy, ClinicalTrials.gov NCT: 01953640) study have been previously published for concurrent solid (metastatic tissue) and liquid (plasma) biopsies before initiating AA/P treatments^18^^,^^20^ and are summarized under Supplementary Methods.

Since both datasets comprise mCRPC biospecimens from patients who had received ADT monotherapy during mHSPC, and in light of evidence from contemporary trials conducted between 2010 and 2020^21-25^ that have established intensified ADT as the current standard of care in mHSPC, we determined the impact of ADT intensification on CNA prevalence in mCRPC biospecimen. For this, we used publicly available datasets in cBioPortal (https://www.cbioportal.org/)^26^^,^^27^ (SU2C/PCF Dream Team cohort [*n* = 444 sequenced samples]^28^). CNA frequencies for the 11 genes of interest in mCRPC samples following ADT intensification were directly downloaded and compared with those from ADT monotherapy as detailed in Supplementary Methods.

### Genomic feature selection and RS construction

Eleven candidate genes were chosen based on previously detailed multigene CNA-based RS.^12^ In brief, after sequencing data were mapped to the reference genome, gene-level read counts were obtained using FeatureCounts with GENCODE Release 19 as the annotation reference.^29^ Gene-specific copy number values were derived by computing log2 ratios of patient read counts relative to the median read counts of normal controls, with thresholds of >0.3 and <−0.3 defining genomic gains and losses, respectively. This yielded copy number changes in 11 survival-associated genes: gains in *AR, MYC, COL22A1, PIK3CA, PIK3CB, NOTCH1* and losses in *TMPRSS2, NCOR1, ZBTB16, TP53, NKX3-1*. The RS was constructed by fitting a multivariate Cox model using the 11 gene-specific CNAs as binary predictors, with the resulting regression coefficients serving as weights in a linear predictor. The RS values were dichotomized at the median into high and low categories for both tissue and ctDNA. Higher RS values indicate a more unfavorable CNA profile.

### Statistical methods

In the YODA COU-AA-302 cohort, univariate Cox regression analyses were first conducted to identify predictors of OS. Variables with significant associations (*P* < .1) and with those considered clinical relevance were then included in a multivariate Cox regression model to estimate adjusted hazard ratios and identify independent predictors of OS. The final multivariate model included treatment group (abiraterone acetate/prednisone [AA/P] vs prednisone [P]), pSA (Prostate Specific Antigen), ALP, LDH, hemoglobin, and albumin. The pSA levels at study enrollment were widely distributed (Table S2) for which the univariate and multivariate models considered pSA levels at study enrollment in independent categorical groups as detailed in Supplementary Methods. To further explore potential treatment-specific effects on survival, stratified Cox regression analyses were conducted separately within the AA/P and placebo arms.

### Development of the clinico-genomic RS

The “PROMOTE” dataset included clinical factors and a 11-gene CNA composite RS generated separately in metastatic tissue biopsies labeled as “SOLID” and concurrently obtained plasma ctDNA specimens labeled as “LIQUID” from treatment-naïve mCRPC patients. Details for calculating the patient’s clinico-genomic RS from CNAs and clinical factors are provided in Supplementary Methods.

### Prognostic nomogram development

The prognostic nomograms were developed separately for the YODA-COU-AA-302 and PROMOTE cohorts using the same statistical approach. The statistical approach for selecting variables at the univariate and multivariate levels is detailed in Supplementary Methods. In each dataset, independent predictors to build the nomogram were based on multivariate Cox proportional hazards analyses, with variables selected on the basis of univariate analysis results and clinical relevance. Regression coefficients from each model were used to assign points to predictors in the corresponding nomogram. Detailed methods for building the nomograms for predicting 1-, 2-, and 3-year OS probabilities for the PROMOTE and the COU-AA-302 cohorts are also provided in Supplementary Methods.

### Nomogram performance and validation

Model performance was evaluated using the time-dependent receiver operating characteristic curves. The time-dependent area under the curve (t-AUC) was computed to evaluate the model’s discriminative ability over different follow-up periods. Internal validation was performed using 1000 iterations bootstrap resampling. All analyses were conducted in RStudio (Version 4.1.2). The survival analysis was applied using “*survival*” package and nomograms was constructed using the “*nomogram*” function from the “*rms*” package. Statistical significance was defined by a 2-sided *P* < .05.

### Statistical analysis of CNA prevalences post-ADT monotherapy vs exposure to androgen-receptor pathway inhibitors (±taxanes)

SU2C/PCF Dream Team samples were stratified into 3 clinical groups based on treatment exposure status as detailed in Supplementary Methods. The 3 groups included a “ADT monotherapy group,” an “Intensified ADT group 1,” and an “Intensified ADT group 2.” Methods for extraction of copy number calls in all 11 genes in all 3 groups and statistical approach for comparisons of CNA prevalences in the 3 groups are detailed in Supplementary Methods.

## Results

Descriptive patient summaries for the 2 mCRPC treatment-naïve cohorts are presented in Table S2 (for “YODA-COU-302”) and Table S3A (for “PROMOTE”). A total of 846 of 1088 patients with complete clinical data included for clinical nomogram development had 462/846 patients treated with AA/P and 384/846 with P alone. In the YODA database, 662 of 846 patients had died at the time of data cutoff for analysis which included 307 of 462 in the AA/P and 315 of 384 in the P-only arm. For the single-arm (AA/P) cohort study (PROMOTE dataset), results of the clinical factors and genomic RSs from the CNAs of the 11 genes observed in the metastatic (“SOLID”) and “liquid” (matched) biopsies before initiating AA/P are provided in Tables S3A and S3B, with 51 and 82 patients dead from disease progression and 31 and 82 alive at the date of analysis (Table S3A). The distribution of the 11-gene CNA-based RS for liquid (plasma ctDNA) and solid (metastatic-tissue) biopsies in the PROMOTE cohort was dichotomized into “high” and “low” at the median for both the solid and liquid biopsies as previously reported^12^^,^^30^ and the OS for these categories is provided in Table S3B. These clinical and prognostic features were included as the final variables for developing the clinical and the clinico-genomic nomogram in the 2 independent datasets.

Tables S4-S7 highlight univariate hazard ratios with 95% confidence intervals for clinical prognostic factors (Table S4; *N* = 846). Table S5 summarizes the univariate hazard ratios with 95% CIs for clinical factors associated with OS in the YODA dataset among patients treated with AA/P (*N* = 462), while Table S6 presents the corresponding univariate hazard ratios (with 95% CIs) for the placebo arm (*N* = 384). Univariate hazard ratios for integrated clinical and RS variable in “PROMOTE” are listed in Table S7 for the solid/metastatic and liquid biopsy sets.

On multivariable analysis (MVA), treatment group (AA/P vs placebo), pSA levels, LDH (high vs low), Hgb, and albumin remained significantly associated with OS with a strong trend toward significance for high vs low ALP. Figures S1-S3 list the multivariate hazard ratios for clinical variables associated with OS for the overall cohort, for the AA/P subcohort, and for the P-alone subcohort, respectively.

For the PROMOTE dataset, both metastatic biopsy–based and liquid biopsy–based integrated clinic-genomic RSs were analyzed for survival. At the univariate analysis, the Cox regression hazard ratios for the clinical variables (pSA, LDH, ALP) and the dichotomized RSs above/below the median (as “high” and “low”) for the (solid) metastatic and the liquid (plasma) PROMOTE dataset are shown in Table S7. At the multivariate Cox regression level, the model considered clinical factors (pSA, ALP, LDH) because of clinical relevance as prognostic factors and the RS (high vs low as dichotomized at the median) for solid and liquid biopsies with OS.

**Figure 1.**
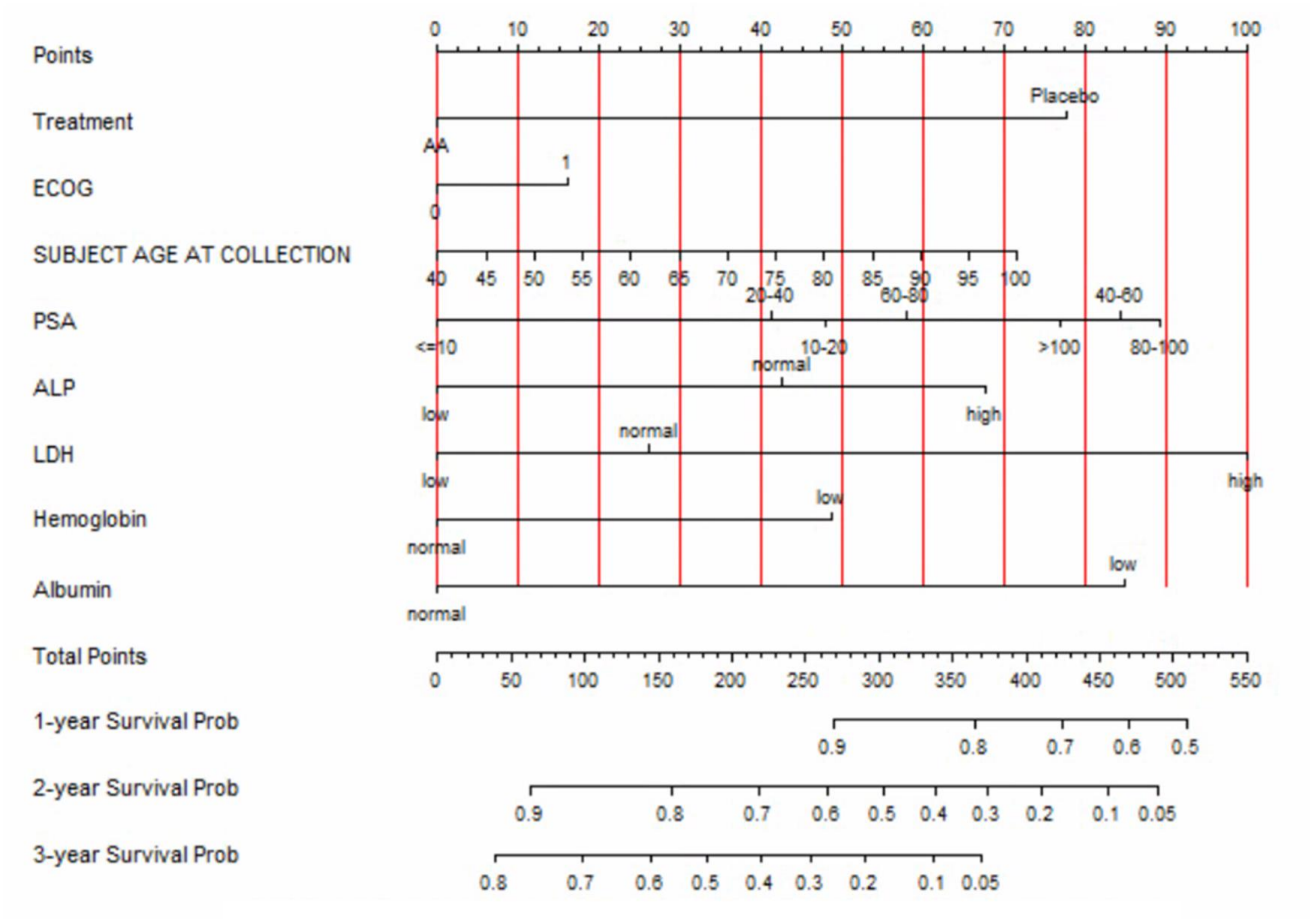
Clinical nomogram for all patients (*N* = 846) in the YODA clinical variable only dataset for estimating overall survival based on treatment group, ECOG performance status, age, PSA level, alkaline phosphatase (ALP), lactate dehydrogenase (LDH), hemoglobin and albumin levels at the time to progression to mCRPC state. Abbreviations: ECOG = Eastern Cooperative Oncology Group; mCRPC = metastatic castrate-resistant prostate cancer; YODA = Yale University Open Data Access.

**Figure 2.**
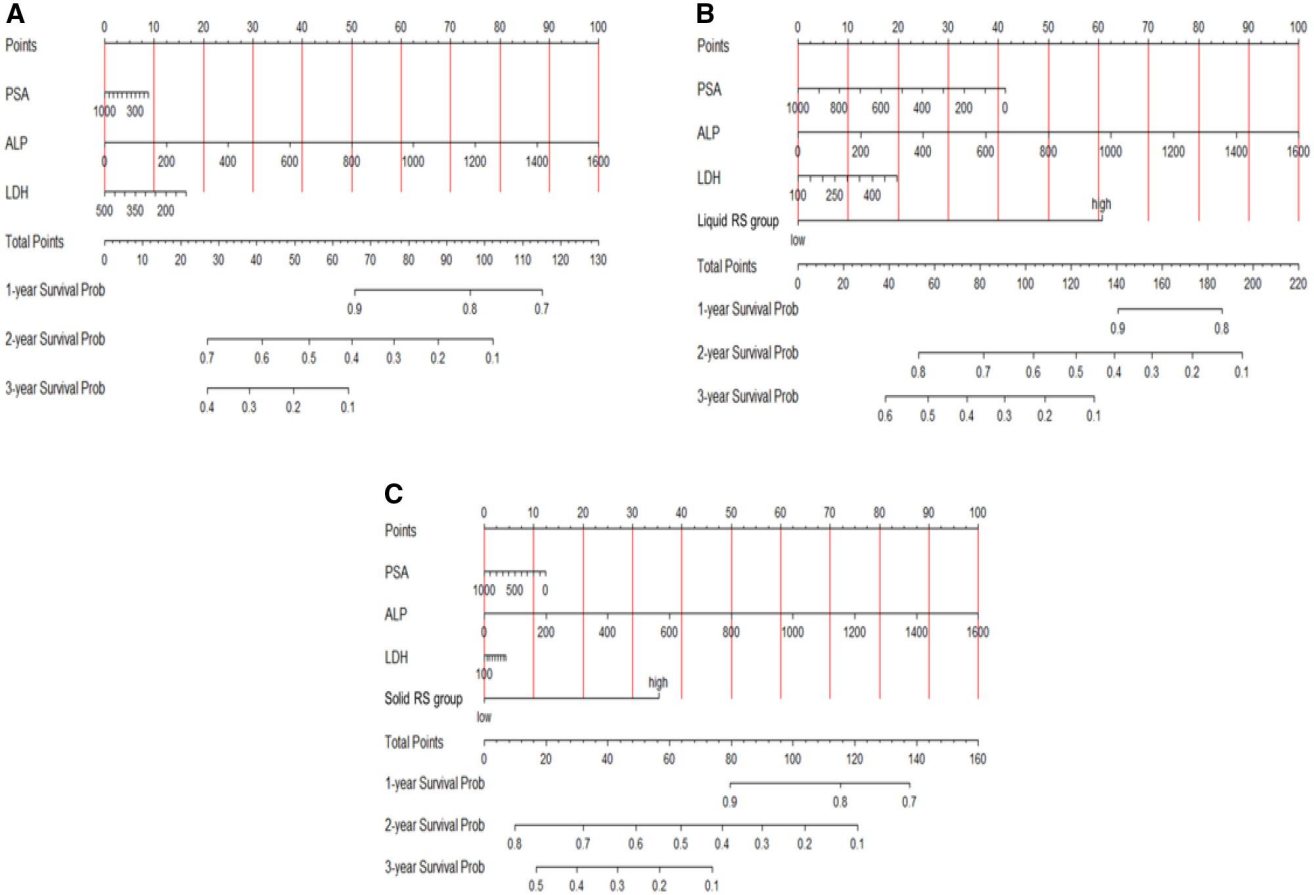
**A**) Clinical-only nomogram (with pSA, LDH, and ALP alone) for the PROMOTE dataset for estimating 1-, 2-, and 3-year survivals. **B**) Clinico-genomic nomogram (integrated liquid plasma ctDNA-biopsy RS groups with pSA, LDH, and ALP) for the “liquid” biopsy PROMOTE dataset for estimating 1-, 2-, and 3-year survivals. **C**) Clinico-genomic nomogram (integrated solid metastatic-biopsy RS groups with pSA, LDH, and ALP) for the “SOLID” metastatic-biopsy PROMOTE dataset for estimating 1-, 2-, and 3-year survivals. Abbreviations: ALP = alkaline phosphatase; LDH = lactate dehydrogenase; RS = risk score.

We constructed survival nomograms for YODA and PROMOTE datasets from the significant MVA hazard ratio coefficients for estimating individual patient survival at 1, 2, and 3 years. Figure 1 shows the clinical nomogram for the YODA dataset for all patients for estimating OS based on treatment group, ECOG performance status, age, pSA level, ALP, LDH, hemoglobin, and albumin levels at the time to progression to mCRPC state, while Figures S4 and S5 demonstrate the clinical nomogram for patients with the same factors who received AA/P and P alone, respectively. The clinical-only nomogram (using pSA, LDH, and ALP alone) for the PROMOTE dataset for estimating 1-, 2-, and 3-year survivals is provided in Figure 2, A and the clinico-genomic integrated nomograms using clinical factors with RS groups for the liquid and the solid are shown in Figure 2, B and C, respectively, for the concurrent 2 biopsy-type PROMOTE datasets.

**Figure 3.**
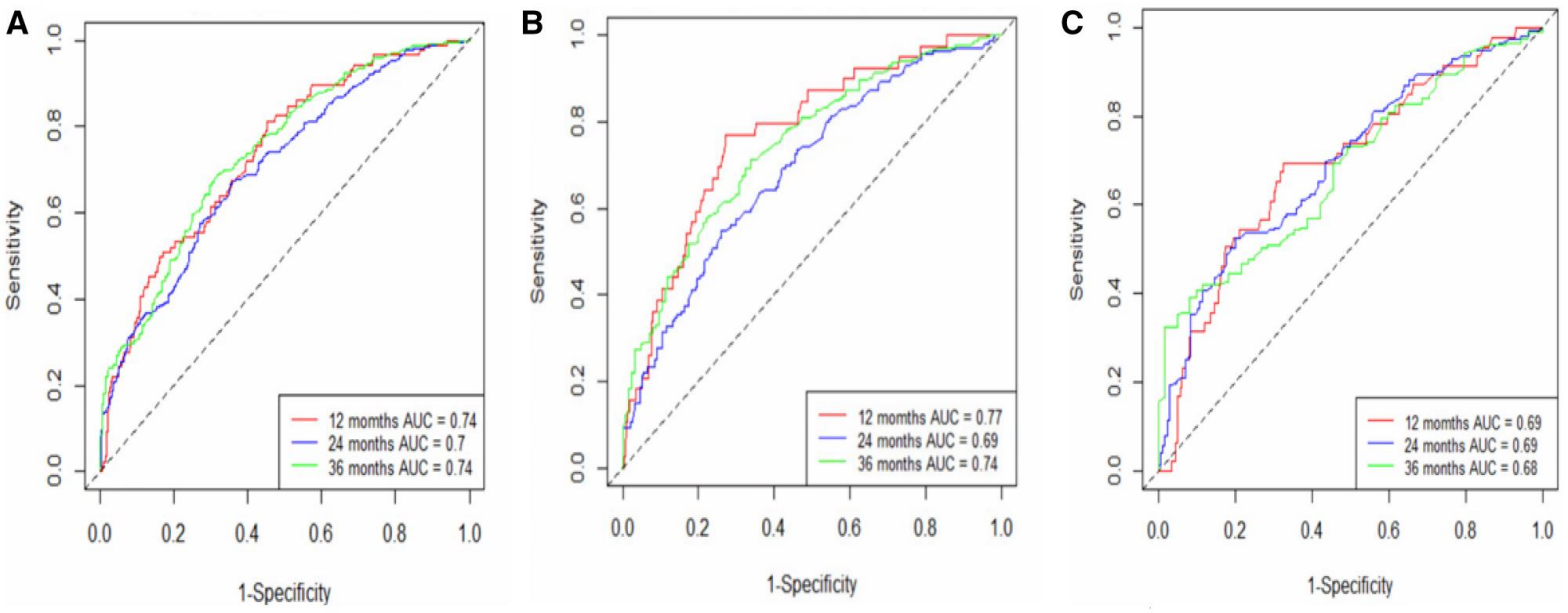
**A**) t-AUC for YODA dataset clinical prognostic nomogram overall survival estimation for all study patients (*N* = 846). **B**) t-AUC for YODA dataset clinical prognostic nomogram overall survival estimation for treatment (AA/P) arm patients (*N* = 462). **C**) t-AUC for YODA dataset clinical prognostic nomogram overall survival estimation for placebo (P) arm patients (*N* = 384). Abbreviations: AA/P = abiraterone acetate/prednisone; t-AUC = time-dependent area under curve; YODA = Yale University Open Data Access.

**Figure 4.**
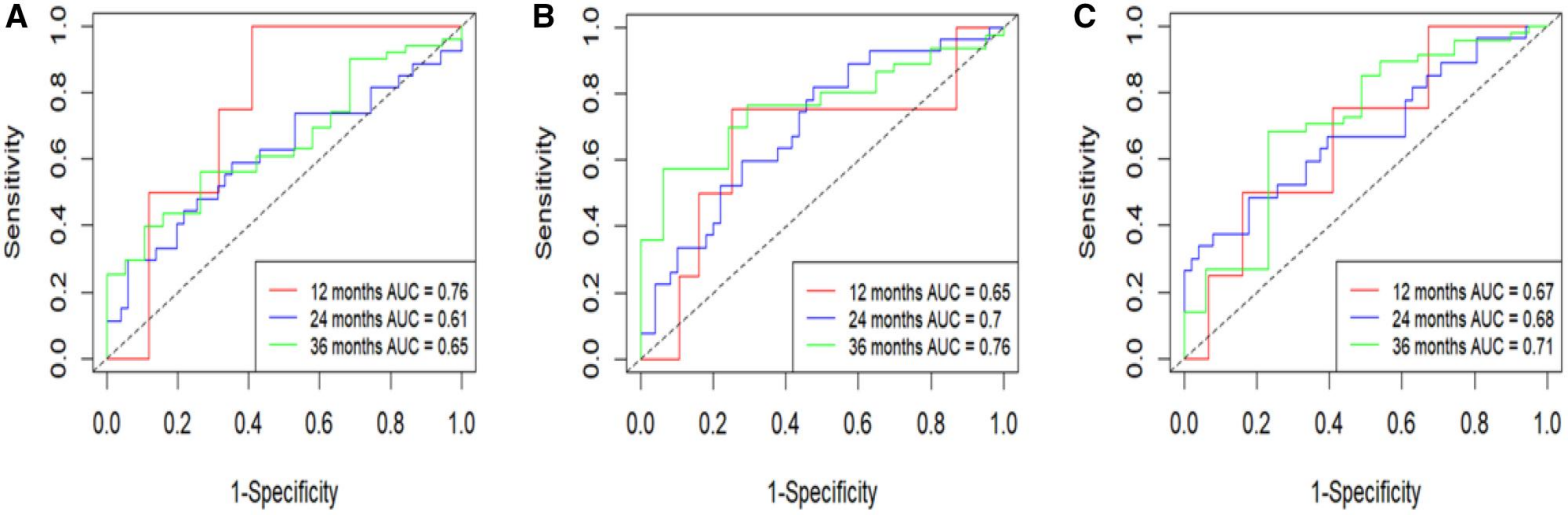
**A**) t-AUC for PROMOTE dataset (*N* = 82) using only the clinical variables (pSA, LDH, ALP) for the prognostic nomogram overall survival estimation. **B**) t-AUC for PROMOTE dataset (*N* = 82) using the integrated (clinical variables—pSA, LDH, ALP with the liquid-biopsy generated 11-gene-based CNA risk score) for the solid-biopsy clinico-genomic prognostic nomogram overall survival estimation. **C**) t-AUC for PROMOTE dataset (*N* = 82) using the integrated (clinical variables—pSA, LDH, ALP with the solid-biopsy generated 11-gene-based CNA risk score) for the liquid-biopsy clinico-genomic prognostic nomogram overall survival estimation. Abbreviations: ALP = alkaline phosphatase; CNA = copy number alteration; LDH = lactate dehydrogenase; t-AUC = time-dependent area under curve.

The performance of the clinical and integrated prognostic nomograms using t-AUC for 1-, 2-, and 3-year OS estimation is shown in Figure 1. A shows the t-AUC of the YODA dataset (*N* = 864) for the clinical variable–based prognostic nomogram for the full cohort. Figure 3, B and C shows the t-AUC for the AA/P and the placebo (P) study arms of the YODA dataset, respectively, for estimating survival using the clinical variable-based prognostic nomogram. Figure 4, A shows the performance characteristics with the t-AUC for the clinical-only prognostic nomogram for the PROMOTE dataset (*N* = 82). Figure 4, B shows the t-AUC for PROMOTE dataset (*N* = 82) using the integrated (clinical variables—pSA, LDH, ALP with the liquid-biopsy 11-gene-based CNA RS) for the prognostic nomogram OS estimation and Figure 4, C similarly shows the t-AUC for PROMOTE dataset (*N* = 82) using the integrated clinical variables—pSA, LDH, ALP with the solid-biopsy generated 11-gene-based CNA RS.

Table 1 presents the overall performance summary for the t-AUCs in the YODA (clinical only) and for the solid and liquid PROMOTE (integrated-clinico-genomic) prognostic nomograms for estimating 1-, 2-, and 3-year OS databases.

**Table 1.** Overall summary of the time-dependent receiver operator curve area under the curve (t-AUC) for OS estimates for the YODA PROMOTE database.

	AUC for 12-month OS estimate	AUC for 24-month OS estimate	AUC for 36-month OS estimate
**YODA (*N* = 846) All study patients**	0.74	0.7	0.74
**YODA (*n* = 462) AA/P treatment arm**	0.77	0.69	0.74
**YODA (*n* = 384) placebo arm**	0.69	0.69	0.68
**Metastatic PROMOTE: Clinical only** **AUC using clinical factors—ALP/LDH/pSA**	0.803	0.612	0.694
**Metastatic PROMOTE: Integrated** **AUC using ALP/LDH/pSA and 11-gene RS**	0.664	0.662	0.714
**Liquid-biopsy-based PROMOTE: Clinical only** **AUC using clinical factors—ALP/LDH/pSA**	0.599	0.593	0.629
**Liquid-biopsy-based PROMOTE: Integrated** **ctDNA AUC using ALP/LDH/pSA and 11-gene RS**	0.649	0.654	0.807

Abbreviations: AA/P = abiraterone acetate/prednisone; ALP = alkaline phosphatase; CNA = copy number alteration; ctDNA = circulating tumor DNA; LDH = lactate dehydrogenase; OS = overall survival; RS = risk score; YODA = Yale University Open Data Access.

Since the YODA and PROMOTE mCRPC datasets were collected after patients were exposed only to ADT monotherapy during mHSPC stage, we evaluated the prevalence of CNAs across 11 genes in mCRPC biospecimens in the SU2C/PCF dataset^28^ where mCRPC samples have also been obtained after exposure to intensified ADT regimens incorporating androgen-receptor pathway inhibitors (ARPIs) and/or taxane therapies. The objective was to identify treatment-associated differences in CNA prevalence consistent with therapy-induced lineage plasticity and to assess the generalizability of this prognostic model in contemporary mHSPC patient populations receiving intensified ADT.

In the SU2C/PCF Dream Team datasets, comprehensive sequencing and clinical data are available from 444 mCRPC biospecimens collected following treatment with either ADT monotherapy or intensified ADT regimens incorporating ARPIs; AA/P or enzalutamide, with or without taxane chemotherapy. Figure S6, A summarizes the subset of patients (*N* = 180/444) with prior ADT-based treatment exposure in cBioPortal-based SU2C/PCF database. Figure S6, B details the therapeutic regimen distribution preceding specimen collection. Figure S6, C compares the fraction of genome altered (FGA), calculated from global CNAs, between patients treated with ADT monotherapy and those exposed to intensified ADT regimens, demonstrating no significant differences in FGA across treatment groups prior to mCRPC biopsy. Figure S6, D evaluates differences in the prevalence of the 11 CNAs across treatment groups, demonstrating no significant differences for 10 of the 11 CNAs, with a statistically significant increase in the prevalence of a single CNA (AR gain) observed in patients previously treated with intensified ADT compared with ADT monotherapy alone.

## Discussion

Current prognostic approaches for survival estimation in mCRPC rely on stratifying patients into broad risk subgroups (Table S1), an approach that inherently limits the precision of patient-level survival predictions. Molecular biomarkers in the mCRPC setting for prognostication or treatment effect prediction have largely been limited to single-biomarker analyses. We identified 79 contemporary studies summarized in Figure S7 and Table S8, reporting individual molecular alterations for prognostic or predictive purposes, several of which include studies conducted retrospectively. None of these studies include an algorithmic framework that integrates molecular biomarkers with clinical variables for clinical application, except in 1 study where pathogenic genetic alterations and clinical factors were integrated into clinical-genetic models for categorizing high vs low risk categories of survival,^31^ without individualizing the estimates using a nomogram approach. In this study, we developed patient-centric prognostic nomograms integrating clinical variables with genomic biomarkers, building on our prior 11-gene CNA survival RS in mCRPC derived from metastatic tissue or plasma ctDNA.^12^^,^^30^ The integrated nomograms achieved either comparable for 1 and 2 years or higher discriminative accuracy than clinical-only models for 3-year OS estimation, with AUC increasing from 0.694 (clinical only) to 0.807 for liquid-biopsy-based integrated estimates.

Obtaining plasma ctDNA-based candidate molecular prognostic factors can be achieved in the same blood sample as the nonspecific clinical prognostic factors and an integrated clinico-genomic approach appears feasible and potentially provides more robust patient-centric survival estimates using the nomogram approach. Additionally, the clinico-genomic nomogram approach supports personalized medicine by tailoring predictions to the individual patient’s mCRPC lethality based on tumor biology. It advances the potential for precision targeting of high-risk mCRPC patients destined to have short survival by developing targeted therapies for poor prognostic subcohorts based on altered genomic pathways. Integrating such a classifier strategy has been successfully applied in many other tumor types including lymphoma, myelodysplastic syndrome, multiple myeloma, and other tumor types.^19^

Since the 11-gene RS dataset only included patients with exposure to ADT monotherapy, we evaluated the effect of intensified ADT on the molecular landscape and observed that global somatic CNAs and specifically the prevalence of the 11 CNAs in mCRPC biospecimens from patients previously treated with ADT monotherapy or with intensified ADT regimens do not change. The stability of CNA prevalence following intensified ADT exposure suggests that the RS retains prognostic utility in mCRPC irrespective of the specific ADT regimen administered during the mHSPC stage.

Our study represents an initial step toward improving mCRPC survival prognostication, but needs prospective validation. The study has several limitations including the small sample size of the second (PROMOTE) dataset, while unique in having matched solid-liquid biopsies for developing integrated nomograms with the clinical and genomic prognostic RSs. In addition, the clinical factors in the integrated nomogram used pSA, LDH, and alkaline phosphatase levels only as access to other prognostic factors such as use of opioids, albumin, and hemoglobin levels were not available in the publicly accessed database. These too will need to be evaluated in large treatment-naïve mCRPC datasets, which can develop composite genomic prognostic risk signatures in easier-to-obtain blood samples.

Finally, the overall number of concordant solid-liquid biopsy pairs in this dataset is limited, reflecting the practical challenges of prospectively obtaining such matched samples. Although we addressed potential model overfitting through internal validation using 1000 bootstrap resampling iterations, larger cohorts of patients with matched solid and liquid biopsies and adequate follow-up will be necessary to further validate and strengthen these findings. Despite these limitations, we observed that our initial attempt to develop integrated clinico-genomic nomogram approaches, whether based on metastatic tissue-derived RSs or concurrently obtained ctDNA-derived RSs consistently demonstrated a higher accuracy in estimating 1-, 2-, and 3-year survival probabilities. This supports a potential promising strategy for incorporating such classifiers into biomarker enrichment clinical trial designs,^32^ for poor-risk, treatment-naïve mCRPC patients.

## Supplementary Material

pkag042_Supplementary_Data

## Data Availability

Raw data used for generating the risk scores in the course of the study are available on dbGaP (phs001141; PRJNA325181) and cBioPortal PRAD SU2C datasets (https://cbioportal-datahub.s3.amazonaws.com/prad_su2c_2019.tar.gz). Analysis scripts are available at https://github.com/HuntsmanCancerInstitute/Workflows/ and https://github.com/zakiF/PublishedPapers/ProstatePROMOTE.
