# Supplementary material for: Clinico-genomic nomograms for estimation of survival risk in metastatic castrate-resistant prostate cancer
Source: JNCI Cancer Spectr. 2026 Jun 3;10(3):pkag042. doi: 10.1093/jncics/pkag042 (PMC13231001; doi:10.1093/jncics/pkag042)
Supplement: pkag042_Supplementary_Data [file pkag042_supplementary_data.docx]

**Supplementary Methods**

*Description of the Treatment-naïve mCRPC YODA COU-AA-302 Cohort*

The COUGAR-302 study was a phase 3, randomized, double-blind, placebo-controlled trial sponsored by Janssen Research & Development and conducted between February 2009 and December 2013 [1]. It evaluated the efficacy of abiraterone acetate (AA/P) plus prednisone versus placebo plus prednisone (P) in chemotherapy-naïve men with mCRPC. Eligible participants were asymptomatic or mildly symptomatic, had pathologically confirmed prostate cancer, had not received prior chemotherapy, and had an Eastern Cooperative Oncology Group (ECOG) performance status of 0 or 1.

Data used to develop clinical nomograms from the “YODA COU-AA-302” study were obtained through the Yale Open Data Access (YODA) platform, which provides access to de-identified clinical trial data for scientific analysis. Briefly, the COU-AA-302 study enrolled 1,088 patients randomized to receive Abiraterone Acetate/Prednisone (“AA/P”) (study arm) versus Prednisone (“P”) alone (control arm). We observed that 93 individuals in the placebo arm crossed over to receive AA/P during the study period. To reduce bias related to treatment crossover, these 93 patients were excluded from the prognostic analysis. Another 149/1088 patients with incomplete data were also not included in the nomogram development. A total of 846 patients were included in the analysis, with 462 in the study arm and 384 in the control arm. Overall survival (OS) was defined as the time from the start of treatment on cycle 1, day 1, to the date of death or last follow-up. Eight clinical variables used for risk-group categorizations [2] and for nomogram development included, i) Patient age at the start of treatment; ii)Treatment assignment (AA/P or Placebo); iii) Eastern Cooperative Oncology Group (ECOG) performance status (0 or 1); and laboratory variables at study entry for iv) hemoglobin; v) albumin; vi) alkaline phosphatase (ALP); vii) lactate dehydrogenase (LDH), and viii) prostate-specific antigen (PSA). Laboratory variables were categorized as “low”, “normal”, or “high" based on study reported normal ranges for hemoglobin (normal range: 13.8-17.2 g/dL), albumin (normal range: 3.5-5.5 g/dL), ALP (normal range: 44-147 U/L), LDH (normal range: 135-225 U/L). Since there was a wide range for PSA at study enrollment, PSA levels (in ng/ml) at the time of enrollment were divided into six categories in the cohort as ≤10, (10-20], (20-40], (40–60], (80–100], and >100 (ng/mL).

*Description of the clinical and molecular dataset: The “PROMOTE” Study Cohort*

Details for mCRPC patient eligibility and recruitment for the single-arm prospective cohort “PROMOTE” (Prostate Cancer Medically-Optimized Genome-Enhanced Therapy, https://clinicaltrials.gov/ identifier NCT # 01953640) study have been previously published [3, 4]. Briefly, all patients underwent concurrent solid (metastatic tissue) and liquid (plasma) biopsies before initiating AA/P treatments and a prognostic 11-gene CNA-based prognostic risk score (RS) was developed for each patient for both types of biopsies [5]. The 11-genes with alterations included in this RS include gains in *AR, MYC, COL22A1, PIK3CA, PIK3CB, NOTCH1* and losses in *TMPRSS2, NCOR1, ZBTB16, TP53, NKX3-1.* Individual patient risk scores for metastatic tissue and liquid biopsy-along with de-identified patient outcomes of 82 mCRPC patients were accessed and downloaded from publicly reported databases and published websites [5, 6]. Overall survival (OS) in this cohort was defined as the time from the start of treatment to death or last follow-up. Patients alive at the end of the observation period were censored. Prior to any access of de-identified patient data from these databases for research use, University of Utah Institutional review Board (IRB) approval for research exemption was obtained (IRB_00135608). No patient contact was performed for this study, and all patient data was accessed as de-identified online data from databases. Study enrollment took place between May 2013 and September 2015 with 82 patients enrolled and prospectively followed until October 31^st^ 2018. Molecular data from the PROMOTE-SOLID [4] and PROMOTE-LIQUID datasets [6] were analyzed, with overall survival (OS). OS was defined as the time from the start of treatment to death or last follow-up. Patients without progression or those lost to follow-up at the time of analysis were censored.

In this study, the PROMOTE-SOLID dataset included variables included pre-AA/P treatment PSA, LDH, ALP, and RS based on solid tissue biopsy. The PROMOTE-LIQUID dataset contained PSA, LDH, ALP, and RS obtained from liquid biopsy. The RS was categorized as “low” or “high” based on the median for the cohort as previously reported [5]. Missing values were assessed and imputed accordingly. The missingness were 4.88% for LDH, 7.32% for ALP, and 17.07% for RS and its categorical group. Continuous variables were imputed using the median, while categorical variables were imputed using the mode.

Patient demographics for the YODA and the PROMOTE cohorts are provided in **Tables S2** and **S3** respectively which summarize patient characteristics and clinical variables. Continuous variables, including PSA, ALP, LDH, and RS, were reported as mean, standard deviation (SD), median, minimum, and maximum values. Comparisons between groups (death vs. no death, progression vs. no progression) were conducted using the Mann–Whitney U test for non-normally distributed variables. The significance threshold was set at p < 0.05. Categorical variables were compared using Pearson’s chi-squared test, and Fisher’s exact test for comparisons with small sample sizes (expected frequency < 5). RS values were categorized as “low” or “high” based on above/below the median for the range of the cohort as described previously [5].

**Sample Size calculations and results for the retrospective datasets:** Events Per Variable (EPV) and Post-hoc power were calculated, to detect whether the sample size was sufficient for the number of variables used in the **retrospective** study.

The sample size for a Cox Proportional Hazards model is driven by the number of events (deaths). For a multivariable Cox model, the Events Per Variable (EPV) rule requires >= 10 events [7] for every independent variable in the model to ensure the model stability. But another study suggested that the EPV should be >=20 [8] to eliminate bias in regression coefficients when many low-prevalence predictors are included in a Cox model.

In PROMOTE study, we therefore assumed an EPV>/=15 and since we have 4 variables (PSA, ALP, LDH, RS-groups) in the integrated nomogram, the number of events (deaths) should be at least 60. The event rate we observed is 51/82 deaths.

Based on the results of our study we make the following observation on the sample size justifications:

**For YODA study:** Post-hoc power analysis was performed to detect clinically meaningful associations. With 622 deaths among 846 patients and 15 predictors (Event per variable analysis, EPV=41.5), more than the minimum cutoff for model stability EPV>=20. the study had >80% power to detect hazard ratios ≥1.29 or ≤0.77 at α=0.05, indicating moderate power for identifying clinically meaningful associations.

**For YODA study AA/P group only:** With 307 deaths among 462 patients and 14 predictors (Event per variable analysis, EPV=21.9), more than the minimum cutoff for model stability EPV>=20. the study had >80% power to detect hazard ratios ≥1.41 or ≤0.71 at α=0.05, indicating moderate power for identifying clinically meaningful associations.

**For YODA study P group only:** With 315 deaths among 384 patients and 13 predictors (Event per variable analysis, EPV=22.5), more than the minimum cutoff for model stability EPV>=20. the study had >80% power to detect hazard ratios ≥1.4 or ≤0.71 at α=0.05, indicating moderate power for identifying clinically meaningful associations

**For PROMOTE study:** With 51 events and 5 predictors (EPV=10.2), the study had >80% power to detect hazard ratios ≥1.9 or ≤0.5 at α=0.05. But the strong associations observed for Solid RS group, and Liquid RS group (HRs of 0.346 and 0.453) were within the detectable range, with power >85% for both predictors. According to Vittinghoff, E., & McCulloch, under these circumstances it is still possible to maintain coverage and bias within acceptable levels despite EPV less than 10 [9]. We also performed internal validation using 1000 iterations bootstrap resampling to address the model's overfitting.

**Calculation of the clinico-genomic risk score:**

Cox Proportional Hazard Regression model was applied to evaluate the association between clinical factors (PSA, LDH, ALP), and the 11-gene CNA-RS and OS for both “SOLID” and LIQUID” biospecimens. All clinical variables with complete data for the cohort were included, regardless of their univariate significance if they were considered clinically important based on clinical relevance or prior study evidence. In the final multivariate Cox proportional hazards model, HRs with 95% confidence intervals (CIs) were reported, with HR > 1 indicating increased risk and HR < 1 indicating reduced risk.

For calculating the patient’s clinico-genomic risk score from CNAs and clinical factors we used the Cox proportional hazards models [10] to fit with overall survival (OS). The outcome and each of the potential prognostic risk predictors (change/no change in copy number for individual genes, fraction of upper limit of normal for PSA, ALP and LDH) was taken as the explanatory variable. Then, each patient’s overall risk score is calculated as:

$\mathrm{su}m_{k} \left[ X_{k}*\log HR_{k} \right]$,

where $X_{k}$ denotes the value for the $k^{\mathrm{th}}$ risk predictor (1 or 0 for change/no change in copy number for the specific gene or fraction of upper limit of normal for PSA, ALP and LDH) for the specific patient and $\log HR_{k}$ is the (natural) logarithm of the hazard ratio (i.e., the coefficient) for the $k^{\mathrm{th}}$ risk predictor from the Cox model described above.

**Methods for construction prognostic nomograms**

The prognostic nomograms were developed separately for the YODA-**COU-AA-302** and **PROMOTE** cohorts using the same statistical approach. The statistical approach for selecting variables at the univariate and multivariate levels.

In the YODA **COU-AA-302** cohort the final model included treatment groups (AA/P vs. P), PSA, ALP, LDH, hemoglobin, and albumin with the clinical outcome of OS. Multivariable Cox Regression analysis for all YODA database patients (N=846) was performed including treatment group, ECOG performance Status, ALP (high versus low), LDH (high versus low), Hgb (high versus low), Albumin (high versus low), age (Continuous variable) and the PSA group categories. High and Low categories for all clinical variables were dichotomized at the median for the range observed for the variable. The final model for the PROMOTE dataset included the RS group (low vs. high, dichotomized at the median for the cohorts as previously reported [5]) and clinical variables PSA, ALP, and LDH.

Each predictor was represented on a separate axis, with its associated point scale determined by the regression coefficient in the Cox model. For each individual patient in the dataset, the value of each predictor was located on its axis, a vertical line was drawn towards the *‘points’* scale at the top of the nomogram to determine its score. The scores from all predictors were then added the points from each predictor to get a total points score, which was located on the *‘Total Points’* axis. From the point on the *‘total points’* scale, a vertical line was drawn to the survival probability scales where the intersection indicates the patient’s predicted probability of OS (or PFS) for 1,2 and 3 years. Higher total scores indicated lower predicted survival probabilities.

**Statistical Analysis of CNA Prevalences post ADT monotherapy vs exposure to Androgen-Receptor Pathway Inhibitors (ARPIs) (± Taxanes)**

SU2C/PCF Dream Team samples with adenocarcinoma histology and sequencing results in the cBioPortal (<https://www.cbioportal.org/>) were accessed for determining differences in CNA prevalences between previous ADT monotherapy versus intensified ADT exposure. The clinical groups include:

1) **ADT monotherapy group**: Patients in SU2C/PCF databases who were only treated with ADT monotherapy before metastatic mCRPC tissue biopsy and sequencing and naive to both abiraterone/enzalutamide and taxane-based chemotherapy.

2) **Intensified ADT Group 1**: Patients in SU2C/PCF databases who had documented exposure to abiraterone and/or enzalutamide with ADT before mCRPC metastatic tissue specimen sequencing, regardless of taxane exposure status.

3) **Intensified ADT Group 2**: Patients in SU2C/PCF databases who had documented exposure to abiraterone and/or enzalutamide and taxane-based chemotherapy with ADT before mCRPC metastatic tissue.

We extracted the copy number calls in all 11 genes of interest using gene-specific criteria from all samples in the three clinical groups. For *AR, MYC, NOTCH1, FOXA1, COL22A1*, and *BRAF*, samples were classified as altered if the copy number value was ≥ +1, corresponding to gain or amplification. All other values (0, −1, −2) were classified as not altered. For *TMPRSS2, NCOR1, ZBTB18, TP53*, and *NKX3-1*, samples were classified as altered if the copy number value was ≤ −1, corresponding to heterozygous or homozygous deletion. All other values (0, +1, +2) were classified as not altered.

Fisher’s exact test was used to compare CNA frequencies between the ADT monotherapy Group 1 and SUC/PCF Groups 2/3. For each gene, alteration status (altered vs. not altered) was compared between the two groups using a contingency table. To account for multiple hypothesis testing across the 11 genes analyzed, p-values were adjusted using the Benjamini-Hochberg false discovery rate (FDR) method. Statistical significance was defined as an adjusted p-value < 0.05.

In order to determine the impact on genomic alterations after exposure to ADT monotherapy, and group 2 and for group 3 the fraction genome altered (FGA) in the specimens collected after these treatments was also compared across the different treatment groups. FGA was defined as the proportion of the genome affected by copy number alterations and was obtained from clinical annotation data provided in the SUC/PCF cBioPortal database. Comparisons for CNA prevalence between the groups post ADT monotherapy and the “Group 1 and 2” were performed using the non-parametric Wilcoxon rank-sum test.

**Handling of Missing data in the two retrospective datasets:**

For YODA study, we exclude observations with any missing data from this randomized controlled trial. Missing value included missing time to event, and missing lab variables.

For PROMOTE, the missingness for ALK is 7.3%, LDH is 4.9%, for solid RS and solid RS group, the missingness is 17.1%. Missing data was imputed using multiple imputation chained equations (MICE) with 5 imputations. In MICE, continuous variables (ALK, LDH, solid RS) were imputed using predictive mean matching (PMM), and categorical variables (Solid RS group) were imputed using logistic regression.

CONSORT flow diagrams for the two datasets are being provided below.

Figure for Flowchart for YODA study


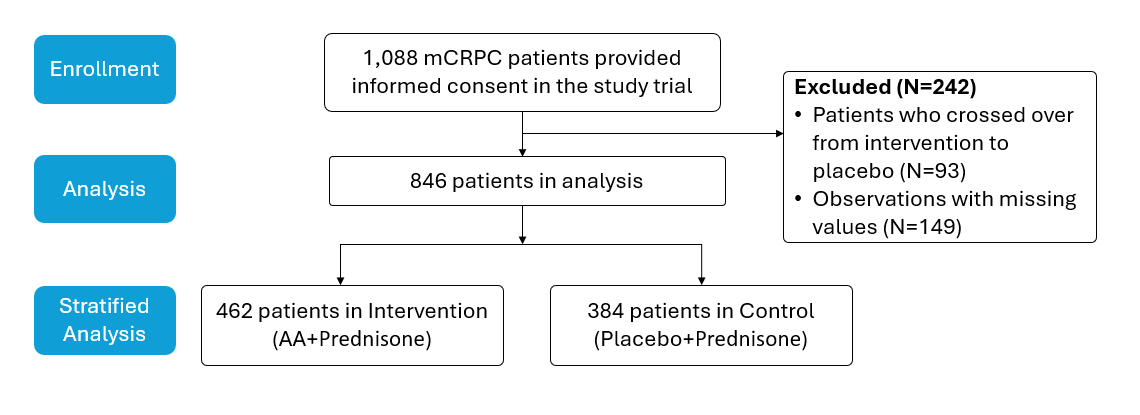


Figure for Flowchart for PROMOTE study


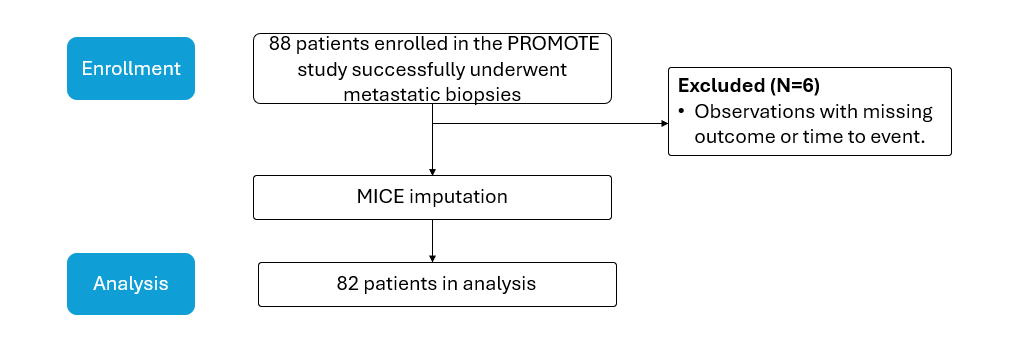


**Supplementary Tables Legend**

**Table S1:** List of mCRPC studies with nomogram or risk-group-based prognostic stratification models based on listed clinical features and cohort sizes with references

**Table S2:** Descriptive summary of patient characteristics for the “YODA-COU-302” cohort

**Table S3A**: Descriptive patient statistics using and risk-scores (RS) in PROMOTE metastatic (Solid) and plasma ctDNA (Liquid) biopsies. **Supplementary Table S3B:** 11-gene CNA-based liquid (plasma circulating-DNA) and solid (metastatic-tissue) RS distribution in PROMOTE cohort and overall survivals for the cohort. “High” Versus “Low” dichotomized above and below the median for the cohort

**Table S4:** Univariate regression for overall survival (OS) for clinical variables for all patients (N=864) in the YODA dataset

**Table S5:** Univariate regression for overall survival (OS) for clinical variables for patients treated with AA/P (N=486) in the YODA dataset

**Table S6:** Univariate regression for overall survival (OS) for clinical variables for patients in the Placebo (P) study arm (N=384) in the YODA dataset

**Table S7:** Univariate Cox Regression HRs for the clinical variables (PSA, LDH, ALP) and the dichotomized Risk Scores (RS) above/below the medianfor the observed range (as “High” and “Low) for the (solid) metastatic and the (liquid (plasma) PROMOTE dataset

**Table S8:** List of studies with molecular prognostic and predictive biomarkers in mCRPC with references. **Figure S7** lists the full table as a figure

**Supplementary Figures Legend**

**Figure S1:** Multivariate Cox Model for YODA, for all patients (N=846) for association of clinical factors with overall survival (OS)

**Figure S2**: Multivariate Cox Model for YODA, for AA/P treatment arm (N=462) for association of clinical factors with overall survival (OS)

**Figure S3**: Multivariate Cox Model for YODA for the Placebo Treatment arm (N=384) for association of clinical factors with overall survival (OS)

**Figure S4:** Clinical Nomogram for patients treated with AA/P (N=462) in the YODA dataset for estimating overall survival based on Treatment group, ECOG Performance Status, Age, PSA level, Alkaline Phosphatase (ALP), Lactate Dehydrogenase (LDH), Hemoglobin and Albumin levels at the time to progression to mCRPC state.

**Figure S5:** Clinical Nomogram for all patients (N=384) in the YODA dataset for estimating overall survival based on Treatment group, ECOG Performance Status, Age, PSA level, Alkaline Phosphatase (ALP), Lactate Dehydrogenase (LDH), Hemoglobin and Albumin levels at the time to progression to mCRPC state.

**Figure S6:** Gene Alteration Frequencies in mCPRC specimens receiving androgen deprivation therapy (ADT) monotherapy or intensified ADT with sample collection post ADT regimens in SU2C/PCF Dream Team dataset

**(A)** Schematic of cohort selection from the SU2C/PCF dataset. Of 444 samples, 326 adenocarcinoma cases were identified and stratified by ADT monotherapy (N = 126), (N = 180), or with missing ADT treatment information (N = 20) groups.

**(B)** Composition of the exposed cohort (N = 180). Each square represents one patient. Exposed samples were classified based on prior systemic therapy into three categories: Enzalutamide (Enza) or Abiraterone (Abi) only (N = 70), taxane only (N = 27), or combined Enzalutamide/Abiraterone plus taxane exposure (N = 83). Two exposed cohort definitions were used for downstream analyses: Exposed Cohort 1, consisting of patients treated with Enzalutamide/Abiraterone plus taxane (N = 83), and Exposed Cohort 2, consisting of patients treated with Enzalutamide/Abiraterone regardless of taxane exposure (N = 153).

**(C)** Comparison of fraction genome altered (FGA) between treatment-naive and exposed cohorts. Box plots show the distribution of FGA values for treatment-naive samples compared with Exposed Cohort 1 (left) and Exposed Cohort 2 (right), with individual samples overlaid as points. P-values were calculated using the Wilcoxon rank-sum test.

**(D)** Gene-level copy number alteration frequencies comparing treatment-naive samples with exposed cohorts. Bar plots show the percentage of samples classified as altered for each gene in treatment-naive versus Exposed Cohort 1 (top) and Exposed Cohort 2 (bottom). Statistical significance was assessed using Fisher's exact test for each gene, with p-values adjusted for multiple testing using the Benjamini-Hochberg method. Adjusted p-values are displayed above genes showing significant differences (p < 0.05) between treatment groups.

**Figure S7:** List of studies with molecular prognostic and predictive biomarkers in mCRPC. **Table S8** lists the full references for this figure.

**Table S1**


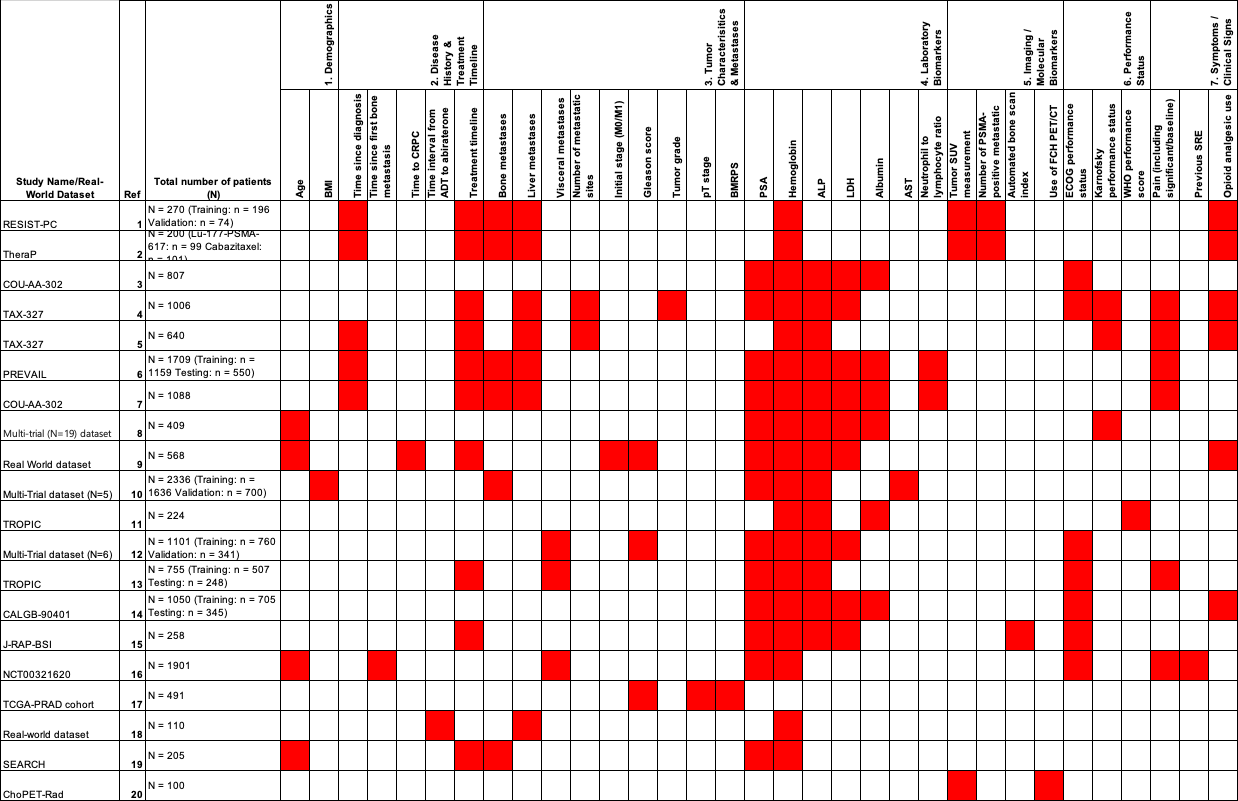


*BMI = Body Mass Index; CRPC = Castrate-Resistant Prostate Cancer ; ADT = Androgen Deprivation Therapy; BMRPS = Bone Metastasis-related Risk Prediction Score; PSA = (Prostate-Specific Antigen); ALP = Alkaline Phosphatase value; LDH = Lactate Dehydrogenase; AST = Aspartate Aminotransferase; SUV = Standardized Uptake Value; FCH PET/CT = 18F-Fluorocholine Positron Emission Tomography/Computed Tomography; ECOG = Eastern Cooperative Oncology Group; WHO = World Health Organization; SRE = Skeletal-related events

**References for Table S1:**

1. Gafita A, Calais J, Grogan TR, Hadaschik B, Wang H, Weber M, Sandhu S, Kratochwil C, Esfandiari R, Tauber R, Zeldin A, Rathke H, Armstrong WR, Robertson A, Thin P, D'Alessandria C, Rettig MB, Delpassand ES, Haberkorn U, Elashoff D, Herrmann K, Czernin J, Hofman MS, Fendler WP, Eiber M. Nomograms to predict outcomes after 177Lu-PSMA therapy in men with metastatic castration-resistant prostate cancer: an international, multicentre, retrospective study. Lancet Oncol. 2021 Aug;22(8):1115-1125. doi: 10.1016/S1470-2045(21)00274-6. Epub 2021 Jul 8. PMID: 34246328.
2. Gafita A, Martin AJ, Emmett L, Eiber M, Iravani A, Fendler WP, Buteau J, Sandhu S, Azad AA, Herrmann K, Stockler MR, Davis ID, Hofman MS. Validation of Prognostic and Predictive Models for Therapeutic Response in Patients Treated with [177Lu]Lu-PSMA-617 Versus Cabazitaxel for Metastatic Castration-resistant Prostate Cancer (TheraP): A Post Hoc Analysis from a Randomised, Open-label, Phase 2 Trial. Eur Urol Oncol. 2025 Feb;8(1):21-28. doi: 10.1016/j.euo.2024.03.009. Epub 2024 Apr 6. PMID: 38584037.
3. Huo X, Kohli M, Finkelstein J. Predicting Survival in Metastatic Castration-Resistant Prostate Cancer Patients: Development of a Prognostic Nomogram. Stud Health Technol Inform. 2025 Apr 8;323:164-168. doi: 10.3233/SHTI250070. PMID: 40200467.
4. Armstrong AJ, Garrett-Mayer ES, Yang YC, de Wit R, Tannock IF, Eisenberger M. A contemporary prognostic nomogram for men with hormone-refractory metastatic prostate cancer: a TAX327 study analysis. Clin Cancer Res. 2007 Nov 1;13(21):6396-403. doi: 10.1158/1078-0432.CCR-07-1036. PMID: 17975152.
5. Armstrong AJ, Garrett-Mayer E, de Wit R, Tannock I, Eisenberger M. Prediction of survival following first-line chemotherapy in men with castration-resistant metastatic prostate cancer. Clin Cancer Res. 2010 Jan 1;16(1):203-11. doi: 10.1158/1078-0432.CCR-09-2514. Epub 2009 Dec 15. PMID: 20008841.
6. Armstrong AJ, Lin P, Higano CS, Sternberg CN, Sonpavde G, Tombal B, Templeton AJ, Fizazi K, Phung D, Wong EK, Krivoshik A, Beer TM. Development and validation of a prognostic model for overall survival in chemotherapy-naïve men with metastatic castration-resistant prostate cancer. Ann Oncol. 2018 Nov 1;29(11):2200-2207. doi: 10.1093/annonc/mdy406. PMID: 30202945; PMCID: PMC6888025.
7. Lorente D, Llacer C, Lozano R, de Velasco G, Romero-Laorden N, Rodrigo M, Sánchez-Iglesias Á, di Capua C, Castro E, Ferrer C, Sánchez-Hernández A, Olmos D. Prognostic Score and Benefit from Abiraterone in First-line Metastatic, Castration-resistant Prostate Cancer. Eur Urol. 2021 Nov;80(5):641-649. doi: 10.1016/j.eururo.2021.07.014. Epub 2021 Aug 6. PMID: 34373138.
8. Smaletz O, Scher HI, Small EJ, Verbel DA, McMillan A, Regan K, Kelly WK, Kattan MW. Nomogram for overall survival of patients with progressive metastatic prostate cancer after castration. J Clin Oncol. 2002 Oct 1;20(19):3972-82. doi: 10.1200/JCO.2002.11.021. PMID: 12351594.
9. Kawahara T, Saigusa Y, Yoneyama S, Kato M, Kojima I, Yamada H, Kamihira O, Tabata K, Tsumura H, Iwamura M, Makiyama K, Uemura H, Miyoshi Y. Development and validation of a survival nomogram and calculator for male patients with metastatic castration-resistant prostate cancer treated with abiraterone acetate and/or enzalutamide. BMC Cancer. 2023 Mar 7;23(1):214. doi: 10.1186/s12885-023-10700-0. PMID: 36882764; PMCID: PMC9990312.
10. Modonutti D, Majdalany SE, Corsi N, Li P, Sood A, Dalela D, Jamil ML, Hwang C, Menon M, Rogers CG, Trinh QD, Novara G, Abdollah F. A novel prognostic model predicting overall survival in patients with metastatic castration-resistant prostate cancer receiving standard chemotherapy: A multi-trial cohort analysis. Prostate. 2022 Sep;82(13):1293-1303. doi: 10.1002/pros.24403. Epub 2022 Jul 5. PMID: 35790016.
11. Belderbos BPS, de Wit R, Hoop EO, Nieuweboer A, Hamberg P, van Alphen RJ, Bergman A, van der Meer N, Bins S, Mathijssen RHJ, van Soest RJ. Prognostic factors in men with metastatic castration-resistant prostate cancer treated with cabazitaxel. Oncotarget. 2017 Nov 16;8(63):106468-106474. doi: 10.18632/oncotarget.22474. PMID: 29290963; PMCID: PMC5739748.
12. Halabi S, Small EJ, Kantoff PW, Kattan MW, Kaplan EB, Dawson NA, Levine EG, Blumenstein BA, Vogelzang NJ. Prognostic model for predicting survival in men with hormone-refractory metastatic prostate cancer. J Clin Oncol. 2003 Apr 1;21(7):1232-7. doi: 10.1200/JCO.2003.06.100. Erratum in: J Clin Oncol. 2004 Aug 15;22(16):3434. PMID: 12663709.
13. Halabi S, Lin CY, Small EJ, Armstrong AJ, Kaplan EB, Petrylak D, Sternberg CN, Shen L, Oudard S, de Bono J, Sartor O. Prognostic model predicting metastatic castration-resistant prostate cancer survival in men treated with second-line chemotherapy. J Natl Cancer Inst. 2013 Nov 20;105(22):1729-37. doi: 10.1093/jnci/djt280. Epub 2013 Oct 17. PMID: 24136890; PMCID: PMC3833929.
14. Halabi S, Lin CY, Kelly WK, Fizazi KS, Moul JW, Kaplan EB, Morris MJ, Small EJ. Updated prognostic model for predicting overall survival in first-line chemotherapy for patients with metastatic castration-resistant prostate cancer. J Clin Oncol. 2014 Mar 1;32(7):671-7. doi: 10.1200/JCO.2013.52.3696. Epub 2014 Jan 21. Erratum in: J Clin Oncol. 2014 May 1;32(13):1387. PMID: 24449231; PMCID: PMC3927736.
15. Kitajima K, Igeta M, Kuyama J, Kawahara T, Suga T, Otani T, Sugawara S, Kono Y, Tamaki Y, Seko-Nitta A, Ishiwata Y, Ito K, Toriihara A, Watanabe S, Hosono M, Miyake H, Yamamoto S, Narita M, Daimon T, Yamakado K. Novel nomogram developed for determining suitability of metastatic castration-resistant prostate cancer patients to receive maximum benefit from radium-223 dichloride treatment-Japanese Ra-223 Therapy in Prostate Cancer using Bone Scan Index (J-RAP-BSI) Trial. Eur J Nucl Med Mol Imaging. 2023 Apr;50(5):1487-1498. doi: 10.1007/s00259-022-06082-3. Epub 2022 Dec 21. PMID: 36539508.
16. Fizazi K, Massard C, Smith M, Rader M, Brown J, Milecki P, Shore N, Oudard S, Karsh L, Carducci M, Damião R, Wang H, Ying W, Goessl C. Bone-related Parameters are the Main Prognostic Factors for Overall Survival in Men with Bone Metastases from Castration-resistant Prostate Cancer. Eur Urol. 2015 Jul;68(1):42-50. doi: 10.1016/j.eururo.2014.10.001. Epub 2014 Oct 29. PMID: 25449207.
17. Luo Y, Deng X, Wei C, Liu Z, Song L, Han K, Li Y, Zhang J, Su S, Wang D. A novel bone metastasis-related gene signature for predicting prognosis, anti-androgen resistance, and drug choice in prostate cancer. J Bone Oncol. 2025 Mar 20;52:100673. doi: 10.1016/j.jbo.2025.100673. PMID: 40226044; PMCID: PMC11986555.
18. Yang YJ, Lin GW, Li GX, Dai B, Ye DW, Wu JL, Xie HY, Zhu Y. External validation and newly development of a nomogram to predict overall survival of abiraterone-treated, castration-resistant patients with metastatic prostate cancer. Asian J Androl. 2018 Mar-Apr;20(2):184-188. doi: 10.4103/aja.aja_39_17. PMID: 29111539; PMCID: PMC5858105.
19. Moreira DM, Howard LE, Sourbeer KN, Amarasekara HS, Chow LC, Cockrell DC, Pratson CL, Hanyok BT, Aronson WJ, Kane CJ, Terris MK, Amling CL, Cooperberg MR, Freedland SJ. Predicting Time From Metastasis to Overall Survival in Castration-Resistant Prostate Cancer: Results From SEARCH. Clin Genitourin Cancer. 2017 Feb;15(1):60-66.e2. doi: 10.1016/j.clgc.2016.08.018. Epub 2016 Aug 31. PMID: 27692812; PMCID: PMC5536956.
20. Cruz-Montijano M, Amo-Salas M, Cassinello-Espinosa J, García-Carbonero I, Villa-Guzman JC, Garcia-Vicente AM. Predictive and Prognostic 18F-Fluorocholine PET/CT Radiomics Nomogram in Patients with Castration-Resistant Prostate Cancer with Bone Metastases Treated with 223Ra. Cancers (Basel). 2024 Jul 29;16(15):2695. doi: 10.3390/cancers16152695. PMID: 39123422; PMCID: PMC11312125.

**Table S2**

|  | **Total Number of Patients (N)** | | | | **Treatment Arm (AA/P)** | | | | **Placebo Arm (P)** | | | |
| --- | --- | --- | --- | --- | --- | --- | --- | --- | --- | --- | --- | --- |
| **Patient Characteristics** | N | Alive  N=224^1^ | Dead  N=622^1^ | p-value^2^ | N | Alive  N=155 | Dead N=307 | p-value^2^ | N | Alive  N=69 | Dead N=315 | p-value^2^ |
| **Planned Treatment Group** | 846 |  |  | <0.001 |  |  |  |  |  |  |  |  |
| AA/P |  | 155 (69%) | 307 (49%) |  |  |  |  |  |  |  |  |  |
| Placebo/P |  | 69 (31%) | 315 (51%) |  |  |  |  |  |  |  |  |  |
| **ECOG** | 846 |  |  | 0.028 | 462 |  |  | 0.021 | 384 |  |  | 0.6 |
| 0 |  | 183 (82%) | 463 (74%) |  |  | 129 (83%) | 226 (74%) |  |  | 54 (78%) | 237 (75%) |  |
| 1 |  | 41 (18%) | 159 (26%) |  |  | 26 (17%) | 81 (26%) |  |  | 15 (22%) | 78 (25%) |  |
| **ALP (IU)** | 846 |  |  | <0.001 | 462 |  |  | <0.001 | 384 |  |  | 0.005 |
| low |  | 12 (5.4%) | 13 (2.1%) |  |  | 7 (4.5%) | 2 (0.7%) |  |  | 5 (7.2%) | 11 (3.5%) |  |
| normal |  | 190 (85%) | 425 (68%) |  |  | 134 (86%) | 209 (68%) |  |  | 56 (81%) | 216 (69%) |  |
| high |  | 22 (9.8%) | 184 (30%) |  |  | 14 (9.0%) | 96 (31%) |  |  | 8 (12%) | 88 (28%) |  |
| **LDH (IU)** | 846 |  |  | <0.001 | 462 |  |  | 0.001 | 384 |  |  | 0.3 |
| low |  | 13 (5.8%) | 23 (3.7%) |  |  | 11 (7.1%) | 16 (5.2%) |  |  | 2 (2.9%) | 7 (2.2%) |  |
| normal |  | 187 (83%) | 458 (74%) |  |  | 131 (85%) | 224 (73%) |  |  | 56 (81%) | 234 (74%) |  |
| high |  | 24 (11%) | 141 (23%) |  |  | 13 (8.4%) | 67 (22%) |  |  | 11 (16%) | 74 (23%) |  |
| **Albumin (gm/dl)** | 846 |  |  | 0.067 | 462 |  |  | 0.019 | 384 |  |  | >0.9 |
| low |  | 12 (5.4%) | 60 (9.6%) |  |  | 7 (4.5%) | 36 (12%) |  |  | 5 (7.2%) | 24 (7.6%) |  |
| normal |  | 212 (95%) | 562 (90%) |  |  | 148 (95%) | 271 (88%) |  |  | 64 (93%) | 291 (92%) |  |
| **Hemoglobin (gm/dl)** | 846 |  |  | 0.007 | 462 |  |  | 0.011 | 384 |  |  | 0.2 |
| low |  | 159 (71%) | 498 (80%) |  |  | 110 (71%) | 251 (82%) |  |  | 49 (71%) | 247 (78%) |  |
| normal |  | 65 (29%) | 124 (20%) |  |  | 45 (29%) | 56 (18%) |  |  | 20 (29%) | 68 (22%) |  |
| **PSA (ng/ml)** | 846 |  |  | <0.001 | 462 |  |  | <0.001 | 384 |  |  | 0.005 |
| <=10 |  | 55 (25%) | 80 (13%) |  |  | 41 (26%) | 38 (12%) |  |  | 14 (20%) | 42 (13%) |  |
| (10-20] |  | 40 (18%) | 74 (12%) |  |  | 25 (16%) | 36 (12%) |  |  | 15 (22%) | 38 (12%) |  |
| (20-40] |  | 51 (23%) | 110 (18%) |  |  | 32 (21%) | 50 (16%) |  |  | 19 (28%) | 60 (19%) |  |
| (40-60] |  | 12 (5.4%) | 55 (8.8%) |  |  | 6 (3.9%) | 28 (9.1%) |  |  | 6 (8.7%) | 27 (8.6%) |  |
| (60-80] |  | 16 (7.1%) | 55 (8.8%) |  |  | 14 (9.0%) | 20 (6.5%) |  |  | 2 (2.9%) | 35 (11%) |  |
| (80-100] |  | 5 (2.2%) | 47 (7.6%) |  |  | 5 (3.2%) | 25 (8.1%) |  |  | 0 (0%) | 22 (7.0%) |  |
| >100 |  | 45 (20%) | 201 (32%) |  |  | 32 (21%) | 110 (36%) |  |  | 13 (19%) | 91 (29%) |  |

^1^ n(%)

^2^ Pearson's Chi-squared test; Fisher's exact test

ALP: Alkaline Phosphatase; LDH: Lactate Dehydrogenase; PSA: Prostate Specific Antigen; ECOG PS: Eastern Cooperative Oncology Group Performance Status; AA/P: Abiraterone Acetate/Prednisone; P: Prednisone/Placebo

For all pts (N=846), 622 (73.5%) patients dead. The median OS month for all is 27.75 (95% CI: 26.30-29.0)

For AA/P group (N=462), 307 (66.5%) patients dead. The median OS month for AA group is 31.86 (95% CI: 28.54-33.90),

For Prednisone/Placebo (P) group (N=384), 315 (82.0%) patients dead. The median OS month for Placebo group is 23.80 (95% CI: 22.12-25.97)

**Table S3A**:

|  | **All (N=82)** | | | **Alive at cut-off date for analysis (N=31)** | **Dead (N=51)** | **P-Value** |
| --- | --- | --- | --- | --- | --- | --- |
|  | Median | Minimum | Maximum | Median | Median |  |
| OS (months) | 25.7 | 3.7 | 51.8 | 34.8 | 23.7 | <0.001 |
| PSA (ng/ml) | 13.5 | 0.4 | 915.0 | 14.6 | 12.3 | 0.44 |
| ALP (IU) | 113.0 | 40.0 | 1588.0 | 100.0 | 128.0 | 0.09 |
| LDH (IU) | 185.0 | 126.0 | 457.0 | 192.0 | 182.0 | 0.43 |
| Solid RS | 0.4 | -0.1 | 4.0 | 0.4 | 0.4 | 0.02 |
| Liquid RS | 0.7 | 0.0 | 5.4 | 0.0 | 0.8 | 0.07 |
| **Table S3B** | | | | | | |

| **Categorical Risk Score (RS) Groups** |  | **Patients Alive** | | **Patients Dead** | | **P-value** |
| --- | --- | --- | --- | --- | --- | --- |
|  | **N** | **N=31** | | **N=51** | |  |
|  |  | N | % | N | % |  |
| **Solid-Biopsy RS group** | 68 |  |  |  |  | 0.11 |
| High RS |  | 7 | 29% | 23 | 52% |  |
| Low RS |  | 17 | 71% | 21 | 48% |  |
| **Liquid-Biopsy RS group** | 82 |  |  |  |  | 0.017 |
| High RS |  | 9 | 29% | 30 | 59% |  |
| Low RS |  | 22 | 71% | 21 | 41% |  |

OS: Overall Survival; RS: Risk Score

ALP: Alkaline Phosphatase; LDH: Lactate Dehydrogenase; PSA: Prostate Specific Antigen; Solid RS: Recurrence Score in solid (metastatic-tissue) biopsies; Liquid RS: Recurrence Score in plasma circulating-DNA-based biopsies

**Table S4:**

| **Clinical variables** | **Hazard Ratio (HR)** | **P-value** | **Lower CI.95** | **UpperCI.95** |
| --- | --- | --- | --- | --- |
| Treatment Group: Placebo vs AA/P | 1.88 | <0.0001 | 1.61 | 2.21 |
| ECOG Performance Status  1 vs 0 | 1.33 | 0.002 | 1.11 | 1.59 |
| Age (years) | 1.02 | 0.0002 | 1.01 | 1.03 |
| ALP normal vs low | 1.56 | 0.1131 | 0.9 | 2.72 |
| ALP high vs low | 2.69 | 0.0006 | 1.53 | 4.74 |
| LDH normal vs low | 1.55 | 0.0412 | 1.02 | 2.36 |
| LDH high vs low | 3.38 | <0.0001 | 2.17 | 5.28 |
| Albumin normal vs low | 0.45 | <0.0001 | 0.34 | 0.59 |
| Hemoglobin normal vs low | 0.69 | 0.0002 | 0.56 | 0.84 |
| PSA 10-20 vs <=10 ng/mL | 1.5 | 0.0121 | 1.09 | 2.06 |
| PSA 20-40 vs <=10 ng/mL | 1.55 | 0.0031 | 1.16 | 2.07 |
| PSA 40-60 vs <=10 ng/mL | 2.01 | 0.0001 | 1.49 | 2.98 |
| PSA 60-80 vs <=10 ng/mL | 2.11 | 0.0001 | 1.42 | 2.84 |
| PSA 80-100 vs <=10 ng/mL | 2.52 | <0.0001 | 1.75 | 3.62 |
| PSA >100 vs <=10 ng/mL | 2.37 | <0.0001 | 1.82 | 3.08 |

ALP: Alkaline Phosphatase; LDH: Lactate Dehydrogenase; PSA: Prostate Specific Antigen; ECOG PS: Eastern Cooperative Oncology Group Performance Status; AA/P: Abiraterone Acetate/Prednisone; P: Prednisone/Placebo

**Table S5:**

| **Clinical variables** | **Hazard Ratio (HR)** | **P-value** | **Lower CI.95** | | | **UpperCI.95** | | |
| --- | --- | --- | --- | --- | --- | --- | --- | --- |
| ECOG 1 vs 0 | 1.4 | 0.009 | 1.09 | | | 1.81 | | |
| Age | 1.02 | 0.0055 | 1.01 | | | 1.03 | | |
| ALP normal vs low | 4.83 | 0.0272 | 1.19 | | | 19.54 | | |
| ALP high vs low | 10.48 | 0.0011 | 2.56 | | | 42.85 | | |
| LDH normal vs low | 1.59 | 0.0752 | 0.95 | | | 2.65 | | |
| LDH high vs low | 3.97 | <0.0001 | 2.28 | | | 6.92 | | |
| Albumin normal vs low | 0.36 | <0.0001 | 0.25 | | | 0.51 | | |
| Hemoglobin normal vs low | 0.64 | 0.0027 | 0.48 | | | 0.86 | | |
| PSA 10-20 vs <=10 | 1.68 | 0.0277 | 1.06 | | | 2.67 | | |
| PSA 20-40 vs <=10 | 1.81 | 0.0066 | 1.18 | | | 2.77 | | |
| PSA 40-60 vs <=10 | 2.54 | 0.0002 | 1.55 | | | 4.16 | | |
| PSA 60-80 vs <=10 | 2.05 | 0.0103 | 1.18 | | | 3.53 | | |
| PSA 80-100 vs <=10 | 2.97 | <0.0001 | 1.78 | | | 4.96 | | |
| PSA >100 vs <=10 | 3.11 | <0.0001 | 2.13 | | | 4.54 | | |
| **Clinical variables** | **Hazard Ratio (HR)** | **P-value** | | **Lower CI.95** | **UpperCI.95** | | UpperCI.95 |  |
| ECOG 1 vs 0 |  | 1.4 | | 0.009 | 1.09 | | 1.81 |  |
| Age |  | 1.02 | | 0.0055 | 1.01 | | 1.03 |  |
| ALP normal vs low |  | 4.83 | | 0.0272 | 1.19 | | 19.54 |  |
| ALP high vs low |  | 10.48 | | 0.0011 | 2.56 | | 42.85 |  |
| LDH normal vs low |  | 1.59 | | 0.0752 | 0.95 | | 2.65 |  |
| LDH high vs low |  | 3.97 | | <0.0001 | 2.28 | | 6.92 |  |
| Albumin normal vs low |  | 0.36 | | <0.0001 | 0.25 | | 0.51 |  |
| Hemoglobin normal vs low |  | 0.64 | | 0.0027 | 0.48 | | 0.86 |  |
| PSA 10-20 vs <=10 |  | 1.68 | | 0.0277 | 1.06 | | 2.67 |  |
| PSA 20-40 vs <=10 |  | 1.81 | | 0.0066 | 1.18 | | 2.77 |  |
| PSA 40-60 vs <=10 |  | 2.54 | | 0.0002 | 1.55 | | 4.16 |  |
| PSA 60-80 vs <=10 |  | 2.05 | | 0.0103 | 1.18 | | 3.53 |  |
| PSA 80-100 vs <=10 |  | 2.97 | | <0.0001 | 1.78 | | 4.96 |  |
| PSA >100 vs <=10 |  | 3.11 | | <0.0001 | 2.13 | | 4.54 |  |

ALP: Alkaline Phosphatase; LDH: Lactate Dehydrogenase; PSA: Prostate Specific Antigen; ECOG PS: Eastern Cooperative Oncology Group Performance Status; AA/P: Abiraterone Acetate/Prednisone; P: Prednisone/Placebo

**Table S6:**

| **Clinical variables** | **Hazard Ratio (HR)** | **P-value** | **Lower CI.95** | **UpperCI.95** |
| --- | --- | --- | --- | --- |
| ECOG 1 vs 0 | 1.24 | 0.1052 | 0.96 | 1.6 |
| Age | 1.02 | 0.0069 | 1.01 | 1.03 |
| ALP normal vs low | 1.11 | 0.7327 | 0.61 | 2.04 |
| ALP high vs low | 1.39 | 0.3032 | 0.74 | 2.61 |
| LDH normal vs low | 0.58 | 0.1528 | 0.27 | 1.23 |
| LDH high vs low | 1.06 | 0.8828 | 0.49 | 2.3 |
| Albumin normal vs low | 0.58 | 0.0101 | 0.38 | 0.88 |
| Hemoglobin normal vs low | 0.69 | 0.0085 | 0.53 | 0.91 |
| PSA 10-20 vs <=10 | 1.3 | 0.2387 | 0.84 | 2.02 |
| PSA 20-40 vs <=10 | 1.15 | 0.4846 | 0.78 | 1.71 |
| PSA 40-60 vs <=10 | 1.61 | 0.0556 | 0.99 | 2.61 |
| PSA 60-80 vs <=10 | 1.58 | 0.0465 | 1.01 | 2.48 |
| PSA 80-100 vs <=10 | 2.2 | 0.0031 | 1.3 | 3.68 |
| PSA >100 vs <=10 | 1.64 | 0.0084 | 1.13 | 2.36 |

ALP: Alkaline Phosphatase; LDH: Lactate Dehydrogenase; PSA: Prostate Specific Antigen; ECOG PS: Eastern Cooperative Oncology Group Performance Status; AA/P: Abiraterone Acetate/Prednisone; P: Prednisone/Placebo

**Table S7**

| **Prognostic Variables** | **HR** | **P value** | **LowerCI.95** | **UpperCI.95** |
| --- | --- | --- | --- | --- |
| PSA | 1.000 | 0.792 | 0.996 | 1.003 |
| ALP | 1.002 | 0.0045 | 1.000 | 1.003 |
| LDH | 0.999 | 0.787 | 0.993 | 1.005 |
| Solid (Metastatic) RS group^1^ Low vs High | 0.453 | 0.005 | 0.260 | 0.792 |
| Liquid (Plasma ctDNA) RS group1 Low vs High | 0.346 | 0.000 | 0.194 | 0.617 |
| Outcome: OS | | | | |

^1^Solid-RS Group and Liquid-RS-group are categorical variables (Low vs. High); RS: Risk Score; OS: Overall Survival

**Figure S1:**


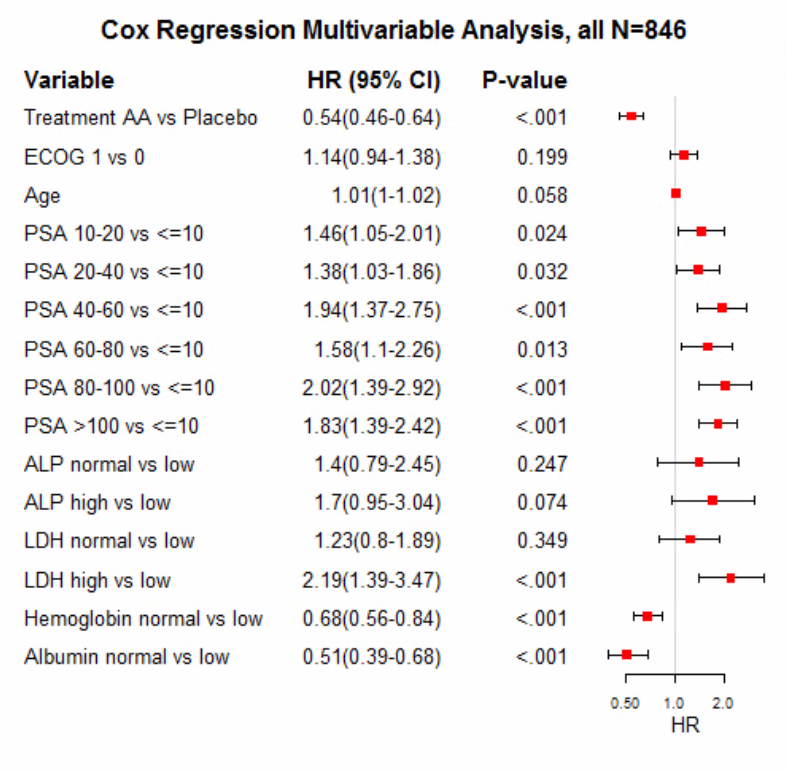


**Figure S2**:


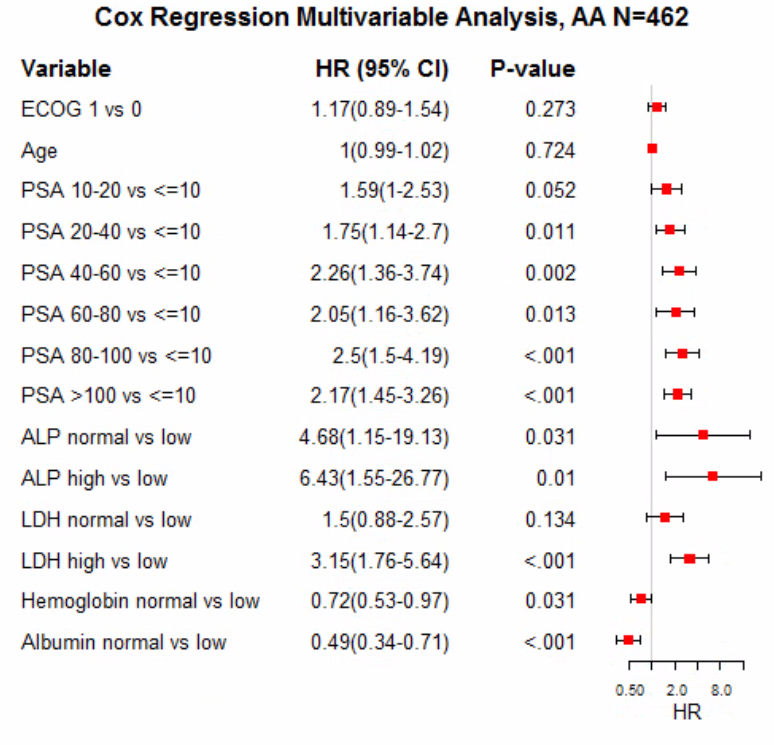


**Figure S3**


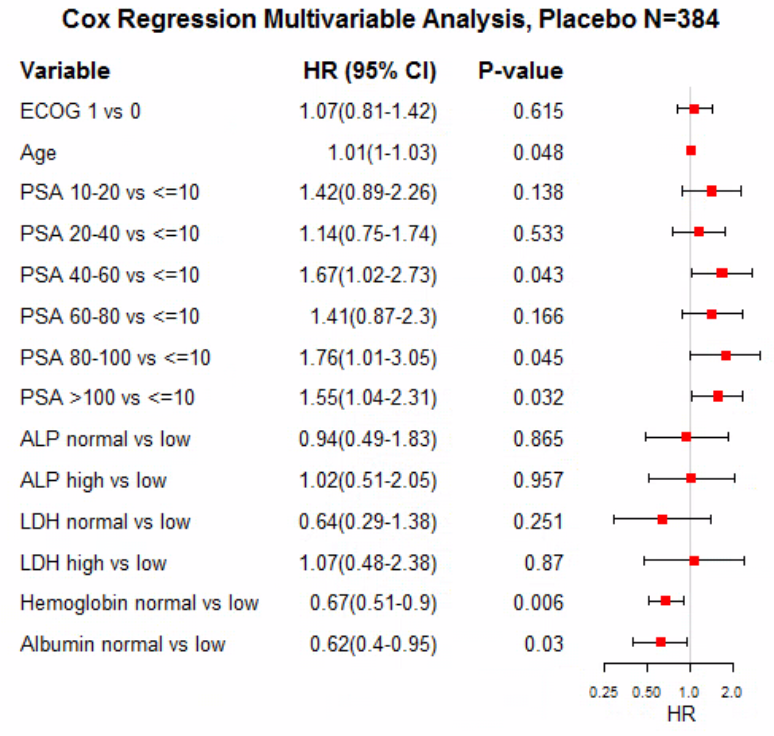


**Figure S4:**


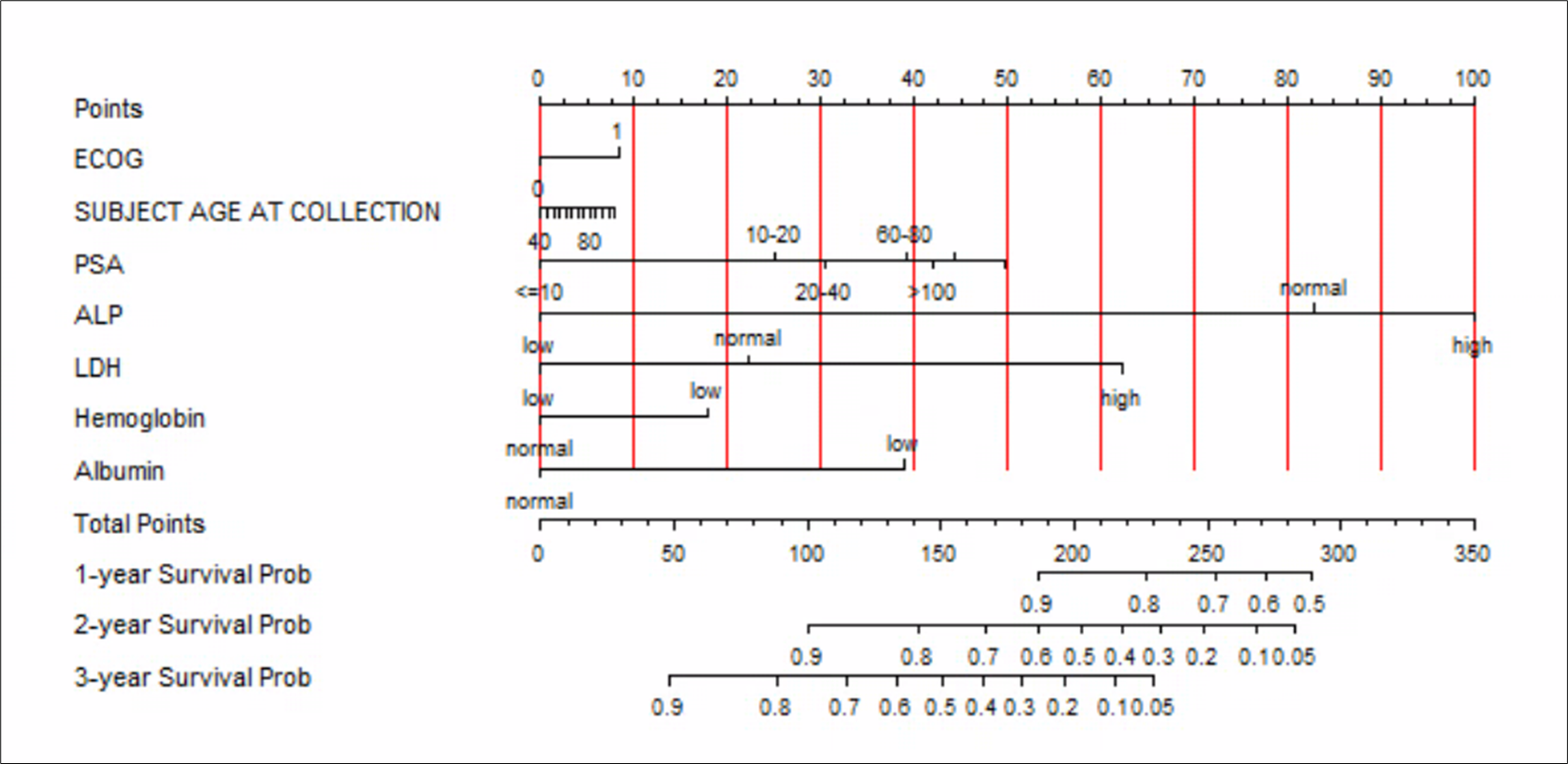


**Figure S5**


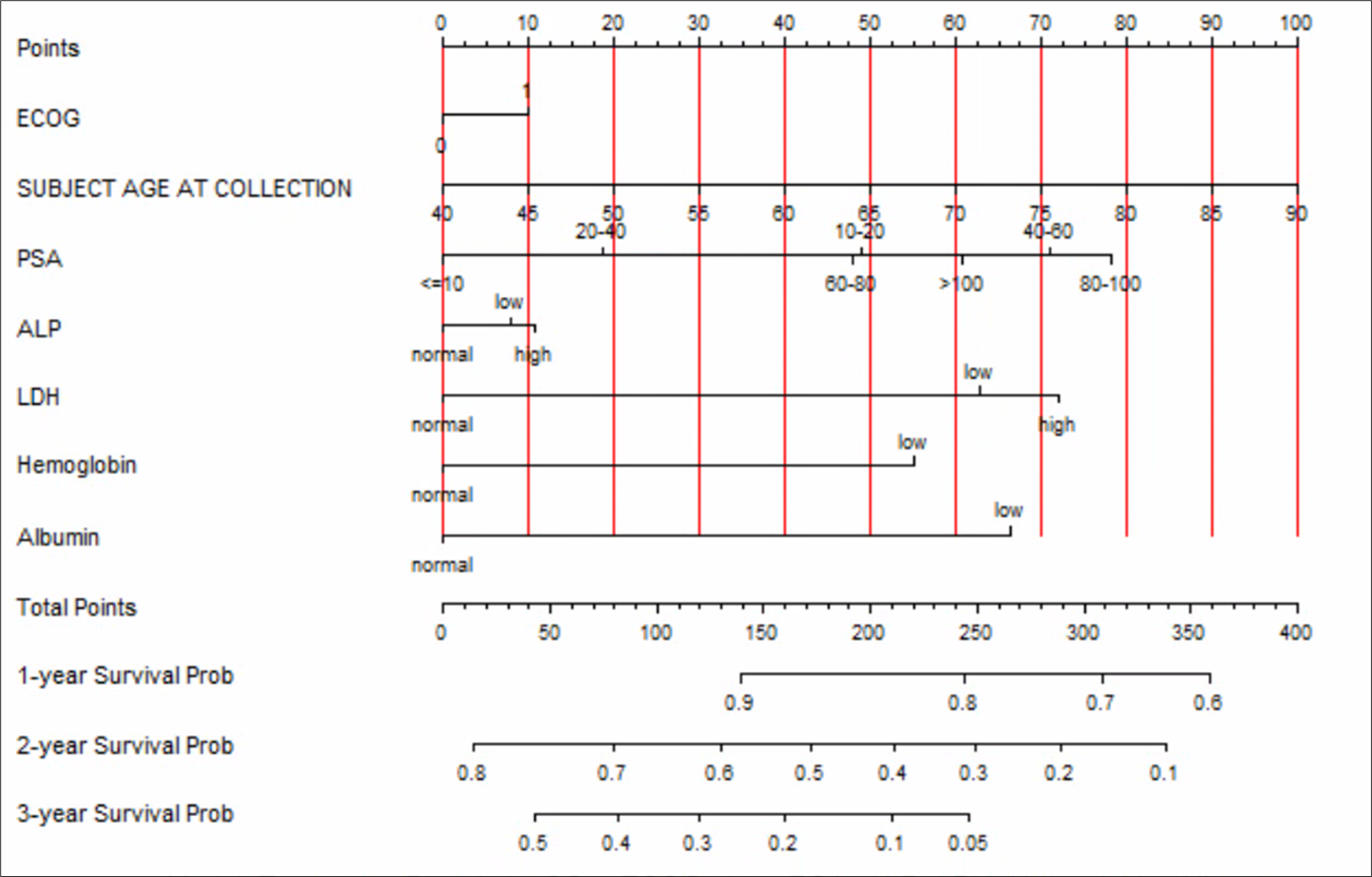


**Figure S6**


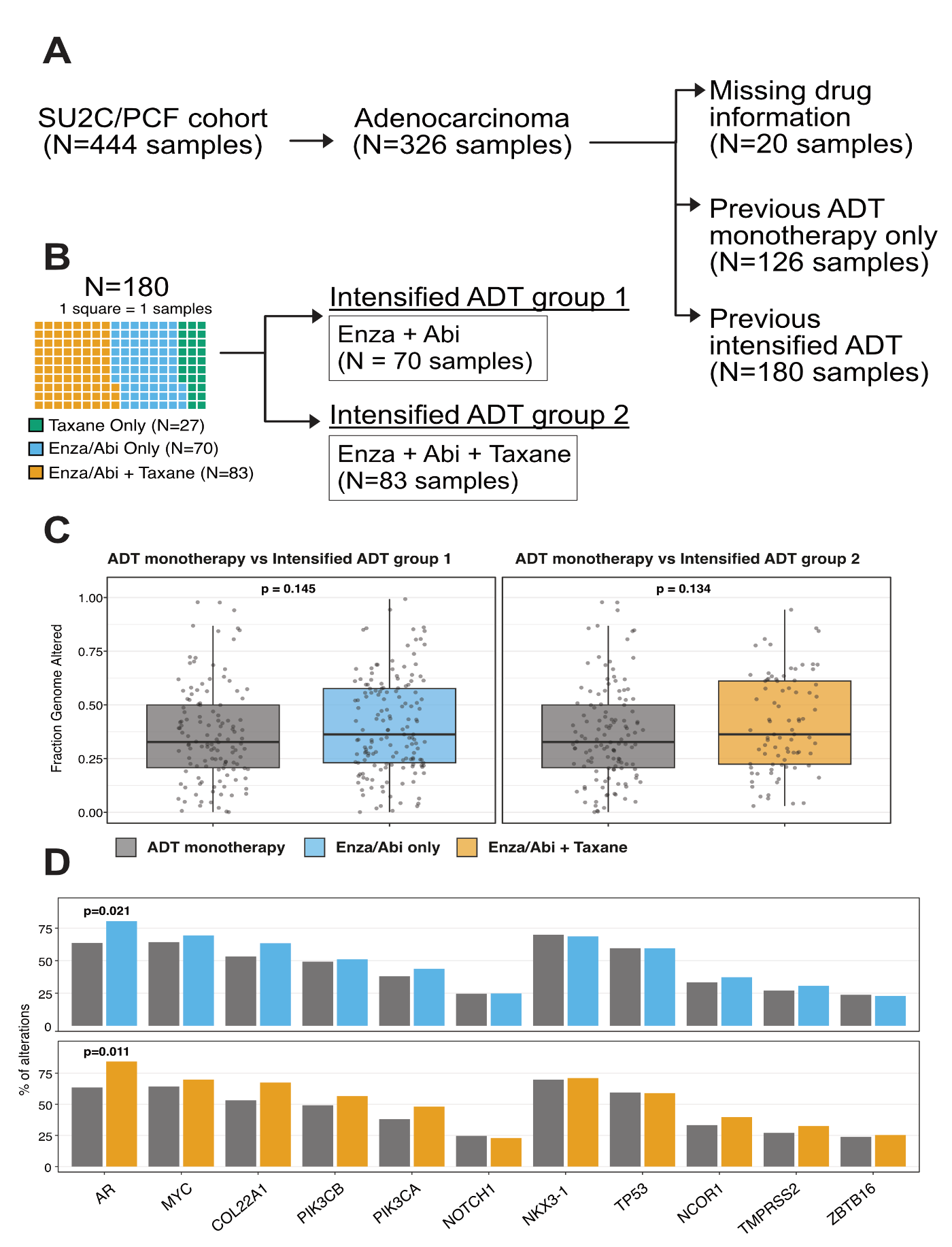


**Figure S7**


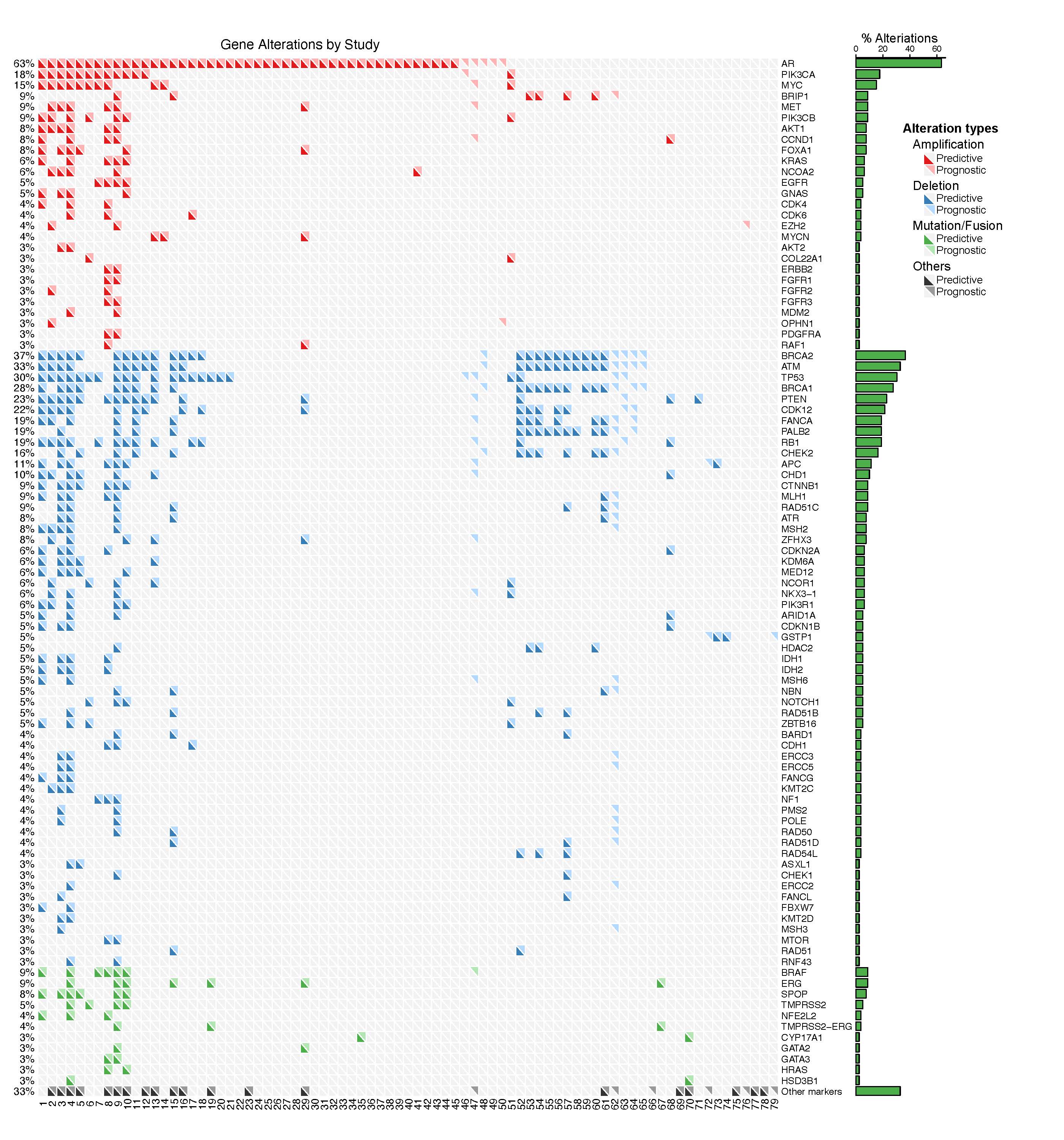


1. Ryan, C.J., et al., *Abiraterone in metastatic prostate cancer without previous chemotherapy.* N Engl J Med, 2013. **368**(2): p. 138-48.

2. Halabi, S., et al., *External Validation of a Prognostic Model of Overall Survival in Men With Chemotherapy-Naive Metastatic Castration-Resistant Prostate Cancer.* J Clin Oncol, 2023. **41**(15): p. 2736-2746.

3. Sicotte, H., et al., *Molecular Profile Changes in Patients with Castrate-Resistant Prostate Cancer Pre- and Post-Abiraterone/Prednisone Treatment.* Mol Cancer Res, 2022. **20**(12): p. 1739-1750.

4. Blee, A.M., et al., *TMPRSS2-ERG Controls Luminal Epithelial Lineage and Antiandrogen Sensitivity in PTEN and TP53-Mutated Prostate Cancer.* Clin Cancer Res, 2018. **24**(18): p. 4551-4565.

5. Fadlullah, M.Z.H., et al., *Multi-gene risk score for prediction of clinical outcomes in treatment-naive metastatic castrate-resistant prostate cancer.* JNCI Cancer Spectr, 2025. **9**(2).

6. Huang, J., et al., *Plasma Copy Number Alteration-Based Prognostic and Predictive Multi-Gene Risk Score in Metastatic Castration-Resistant Prostate Cancer.* Cancers (Basel), 2022. **14**(19).

7. Harrell, F.E., Jr., et al., *Regression models for prognostic prediction: advantages, problems, and suggested solutions.* Cancer Treat Rep, 1985. **69**(10): p. 1071-77.

8. Ogundimu, E.O., D.G. Altman, and G.S. Collins, *Adequate sample size for developing prediction models is not simply related to events per variable.* J Clin Epidemiol, 2016. **76**: p. 175-82.

9. Vittinghoff, E. and C.E. McCulloch, *Relaxing the rule of ten events per variable in logistic and Cox regression.* Am J Epidemiol, 2007. **165**(6): p. 710-8.

10. Cox, D.R., *Regression models and life-tables.* JR stat soc B, 1972. **34**(2): p. 187-220.
